# Breadth and Exclusivity of Hospital and Physician Networks in US Insurance Markets

**DOI:** 10.1001/jamanetworkopen.2020.29419

**Published:** 2020-12-17

**Authors:** John A. Graves, Leonce Nshuti, Jordan Everson, Michael Richards, Melinda Buntin, Sayeh Nikpay, Zilu Zhou, Daniel Polsky

**Affiliations:** 1 Department of Medicine, Vanderbilt University School of Medicine and Vanderbilt University Medical Center, Nashville, Tennessee; 2 Department of Health Policy, Vanderbilt University School of Medicine and Vanderbilt University Medical Center, Nashville, Tennessee; 3 Vanderbilt University School of Medicine and Vanderbilt University Medical Center, Nashville, Tennessee; 4 Department of Health Policy, Vanderbilt University School of Medicine, Nashville, Tennessee; 5 Department of Economics, Baylor University, Waco, Texas; 6 Division of Health Policy and Management, University of Minnesota School of Public Health, Minneapolis; 7 Carey Business School, Bloomberg School of Public Health, Department of Health Policy and Management, Johns Hopkins University, Baltimore, Maryland

## Abstract

**Importance:**

Little is known about the breadth of health care networks or the degree to which different insurers’ networks overlap.

**Objective:**

To quantify network breadth and exclusivity (ie, overlap) among primary care physician (PCP), cardiology, and general acute care hospital networks for employer-based (large group and small group), individually purchased (marketplace), Medicare Advantage (MA), and Medicaid managed care (MMC) plans.

**Design, Setting, and Participants:**

This cross-sectional study included 1192 networks from Vericred. The analytic unit was the network–zip code–clinician type–market, which captured attributes of networks from the perspective of a hypothetical patient seeking access to in-network clinicians or hospitals within a 60-minute drive.

**Exposures:**

Enrollment in a private insurance plan.

**Main Outcomes and Measures:**

Percentage of in-network physicians and/or hospitals within a 60-minute drive from a hypothetical patient in a given zip code (breadth). Number of physicians and/or hospitals within each network that overlapped with other insurers’ networks, expressed as a percentage of the total possible number of shared connections (exclusivity). Descriptive statistics (mean, quantiles) were produced overall and by network breadth category, as follows: extra-small (<10%), small (10%-25%), medium (25%-40%), large (40%-60%), and extra-large (>60%). Networks were analyzed by insurance type, state, and insurance, physician, and/or hospital market concentration level, as measured by the Hirschman-Herfindahl index.

**Results:**

Across all US zip code–network observations, 415 549 of 511 143 large-group PCP networks (81%) were large or extra-large compared with 138 485 of 202 702 MA (68%), 191 918 of 318 082 small-group (60%), 60 425 of 149 841 marketplace (40%), and 21 781 of 66 370 MMC (40%) networks. Large-group employer networks had broader coverage than all other network plans (mean [SD] PCP breadth: large-group employer-based plans, 57.3% [20.1]; small-group employer-based plans, 45.7% [21.4]; marketplace, 36,4% [21.2]; MMC, 32.3% [19.3]; MA, 47.4% [18.3]). MMC networks were the least exclusive (a mean [SD] overlap of 61.3% [10.5] for PCPs, 66.5% [9.8] for cardiology, and 60.2% [12.3] for hospitals). Networks were narrowest (mean [SD] breadth 42.4% [16.9]) and most exclusive (mean [SD] overlap 47.7% [23.0]) in California and broadest (79.9% [16.6]) and least exclusive (71.1% [14.6]) in Nebraska. Rising levels of insurer and market concentration were associated with broader and less exclusive networks. Markets with concentrated primary care and insurance markets had the broadest (median [interquartile range {IQR}], 75.0% [60.0%-83.1%]) and least exclusive (median [IQR], 63.7% [52.4%-73.7%]) primary care networks among large-group commercial plans, while markets with least concentration had the narrowest (median [IQR], 54.6% [46.8%-67.6%]) and most exclusive (median [IQR], 49.4% [41.9%-56.9%]) networks.

**Conclusions and Relevance:**

In this study, narrower health care networks had a relatively large degree of overlap with other networks in the same geographic area, while broader networks were associated with physician, hospital, and insurance market concentration. These results suggest that many patients could switch to a lower-cost, narrow network plan without losing in-network access to their PCP, although future research is needed to assess the implications for care quality and clinical integration across in-network health care professionals and facilities in narrow network plans.

## Key Points

**Question:**

How does the breadth of health care networks and the degree to which they overlap vary within and across specialties and insurance markets?

**Findings:**

In this cross-sectional study of 1192 health care networks, large-group employer networks were broader than small-group employer, marketplace, Medicare Advantage, and Medicaid managed care networks. In many states, narrower networks had as much, if not more, overlap across different insurers’ networks than the broadest networks; areas with less concentrated insurance, physician, and hospital markets had narrower and more exclusive networks.

**Meaning:**

These findings suggest that the structure of plan networks may be a factor in determining care affordability and continuity in the United States, particularly given how frequently individuals change insurance plans.

## Teaser

This cross-sectional study quantifies network breadth and exclusivity (ie, overlap) among primary care physician, cardiology, and general acute care hospital networks for employer-based, individually purchased, Medicare Advantage, and Medicaid managed care plans.

## Introduction

A distinctive trend in US health insurance is narrow networks that limit in-network services to a restricted set of clinicians and facilities.^1,2,3,4,5,6,7,8^ With frequent churn occurring when patients change insurance plans,^9,10,11,12^ insurance network design may have implications for the extent to which churn disrupts the continuity of care. Insurance plan changes decrease the likelihood of establishing a durable primary care relationship, decrease rates of chronic disease control, increase reliance on subspecialists for primary care services, and are associated with greater use of emergency departments.^13,14,15,16^

Given that the risk of care disruptions is higher for beneficiaries in a plan with a narrow network or with a network of physicians who are not likely to be found in other plans, understanding networks along these domains is critical to evaluating their implications for care continuity. While the breadth of networks has been documented in the individually purchased insurance market,^4,5,17^ a more holistic picture of networks is needed to understand implications for care continuity because switching between insurance plans not only occurs within an insurance type but between them. We examined network variation within and between insurance types, including employer-sponsored insurance, Medicare, and Medicaid.

Our study draws on 2019 plan directory data to characterize the breadth and exclusivity—that is, the degree of overlap—of networks in US insurance markets. Using network data for insurers that, collectively, administered plans for approximately three-quarters of individuals with privately administered insurance plans in 2019, we investigated the hypothesis that the size and exclusivity of networks varied across clinician and facility types, states, and the extent of economic market concentration for insurers, physicians, and hospitals.

## Methods

Our study followed the Strengthening the Reporting of Observational Studies in Epidemiology (STROBE) reporting guidelines.^18^ The Vanderbilt University Medical Center institutional review board exempted this study from review and informed consent because no patient data were used, and all data on physicians were publicly accessible based on the National Provider Identifier via the National Plan and Provider Enumeration System.

### Data

We obtained data on physician and hospital insurance network participation from Vericred, a market research firm. Vericred collects network participation data from insurer data feeds and web scrapes of online plan directories. The Vericred data captured information on network participation as of August 2019 for employer-based plans (both self-insured large-group networks and fully insured small-group networks purchased on an exchange), Medicare Advantage (MA) plans, and plans purchased on the Patient Protection and Affordable Care Act marketplace (ie, marketplace) nationwide. In addition, the Vericred data captured Medicaid managed care (MMC) networks as of April 2019.

We used additional data sources to isolate plan networks available in each zip code and to validate information on clinician location and specialty. We used HIX Compare data to identify the geographic markets (health insurance rating area) of marketplace and small-group plans.^19^ We used county-based service area and enrollment files for January 2019 to identify the geographic markets for MA plans (eAppendix in the Supplement).^20^ To isolate service areas of large-group commercial networks and MMC networks, we used 2019 data from Decision Resources Group (DRG). These data contained county-level enrollment (based on enrollee residence) submitted by insurers as part of DRG’s National Proprietary Census. We also used the DRG data to construct measures of insurance market concentration, as described later.

We drew on information on hospital type and geographic location from the 2018 American Hospital Association (AHA) annual survey. To ensure up-to-date specialty and clinic location information for active physicians, we obtained 2019 data from IQVIA and Physician Compare. The IQVIA data captured information on office-based physicians, including their primary specialty and the zip codes of all clinic locations. The Physician Compare data captured information on clinic addresses and primary specialty for all physicians who submitted a Medicare claim within the last 12 months of data collection or who newly registered within the Medicare Provider Enrollment, Chain, and Ownership System (PECOS) within 6 months of data collection.

Finally, our analysis utilized data from the Centers for Medicare & Medicaid Services (CMS) Hospital Service Area files. These files provided summary information on the total number of fee-for-service Medicare patients from each zip code treated at acute care hospitals in 2016 and 2017. We used these data to construct measures of hospital market concentration and to ensure that hospital market definitions for each zip code included acute care facilities used by patients from the zip code.

### Unit of Analysis and Sample Inclusion and Exclusion Criteria

We constructed all measures from the perspective of the patient and/or their referring physician. This approach recognized that an insurance network can be broad from the insurer’s perspective (eg, a network might include >50% of active physicians and hospitals in a state) but narrow from the patient’s perspective (eg, a patient or their referring physician might find that <10% of physicians or hospitals within a 60-minute drive are in-network). To capture the patient perspective, we calculated measures separately by zip code and weighted all analyses by zip code population to yield estimates representative of the US population.^21^

We considered 3 clinical network categories: primary care physician (PCPs; ie, physicians with a primary specialty in internal medicine, general practice, or family practice), cardiology, and general acute care hospitals. We evaluated PCP networks given the predominance of primary care for maintaining coordination and continuity of care, while cardiology and hospital networks captured important high-volume specialty and referral relationships.

We defined a denominator count of the total number of active physicians and hospitals in proximity to each zip code.^22^ We used address information from multiple data sources to geocode and validate the clinic and facility location(s) of active physicians and hospitals (eAppendix in the Supplement). We measured geographic proximity by identifying all active physicians and hospitals within a 60-minute drive of the population-weighted centroid of Zip Code Tabulation Areas, which are geographic representations of zip codes. In sensitivity analyses, we considered a 30-minute drive time for zip codes located within metropolitan core–based statistical areas (ie, nonrural areas). For hospital networks our denominator also included any facility located more than 60 minutes away if at least 2% of fee-for-service Medicare inpatient utilization originating from the zip code was at the hospital. Some zip codes did not have any clinicians or facilities within a 60-minute drive; our results separately report the number and total population within these areas.

In addition to geography and clinical category, we also defined network measures separately by insurance type. Specifically, we considered networks available in a given zip code for (1) large self-insured employer (large group) plans; (2) fully insured small employer plans purchased on an insurance exchange (small group); (3) individually purchased (marketplace) plans; (4) MA plans; and (5) MMC plans. In total, we estimate that our sample captured networks for carriers insuring approximately three-quarters of individuals with privately administered health insurance in 2019 (eAppendix in the Supplement).

Collectively, the previously described criteria meant that our final unit of analysis was the network–zip code–clinician type–insurance type. That is, each observation captured attributes of insurance networks connected with the insurance plans available in each zip code, and based on the set of physicians and hospitals located within a 60-minute drive of the zip code. In total there were 3 868 037 such observations in our data. This overall sample reflected network participation among 220 394 PCPs, 29 512 cardiologists, and 4127 general acute care hospitals within 1192 plan networks available in 32 425 zip codes nationwide.

### Network Size and Exclusivity Measures

Our primary measure of network size was breadth, defined as the percentage of physicians and hospitals located within a 60-minute drive of a hypothetical patient residing in the relevant zip code that were in-network for a given network. Following the literature,^4,23^ we quantified network breadth as a continuous measure and also classified breadth into the following 5 categories: extra-small (<10%), small (10%-25%), medium (25%-40%), large (40%-60%) and extra-large (>60%).

While the breadth measure provided information on the overall size of a network, it did not capture information on the degree of overlap a network had with other insurance carriers’ networks. For example, networks for 2 insurers could be relatively broad but each insurer could have exclusive contracts with physicians and hospitals (ie, there are no overlapping clinicians across the 2 insurers’ networks).

We quantified exclusivity as the percentage of a given network’s physicians and hospitals that overlapped with other carriers’ networks in the same area. This measure was based on the normalized strength of each node in a network of insurance networks (eAppendix in the Supplement). In network analysis methods, normalized strength is defined as the sum of all connections a given node has with other nodes in the network, divided by the total possible number of connections. In the context of our study, each insurance network was a node, and we measured exclusivity as the number of shared physicians and hospitals each network (node) had with other networks (nodes) available in the same zip code. We expressed this value as a percentage by the dividing the total number of shared connections by the total number of possible shared connections and multiplying this value by 100.

Networks with low exclusivity values characterized highly exclusive networks, while those with high values were more connected with other networks. Because the same insurer often offered multiple networks in an area via different plans, we only considered connections with other insurers’ networks. Doing so ensured that insurers with multiple networks in a given area did not receive artificially high exclusivity values simply because their networks had significant overlap with each other; however, we considered the total number of connections in sensitivity analyses.

### Additional Measures

We drew on Hirschman-Herfindahl index (HHI) measures to quantify market concentration within physician, hospital, and insurance markets.^24,25^ Highly concentrated markets are those where the insurer and/or the health care group can exert greater leverage in network inclusion negotiations because they have a large share of enrollment and/or patients. We calculated HHI as the sum of the squared market shares (expressed as a percentage) within markets defined by 625 commuting zones nationwide (eAppendix in the Supplement). For example, a market dominated by a single participant with 100% market share received an HHI value of 10 000 (100^2^) while a market characterized by a large number of participants with similar market shares would receive a low HHI value. Following Department of Justice guidelines,^26^ we classified markets with HHI scores less than 1500 as not concentrated those with scores between 1501 and 2500 as moderately concentrated, and those with scores between 2500 and 10 000 as concentrated.

### Statistical Analysis

We produced descriptive statistics (mean and SD, median and interquartile range [IQR], and quantiles) to summarize the breadth and exclusivity of networks. We used nonparametric Kruksal-Wallis tests for statistical comparisons (α = .05, 2-sided tests) of continuous network breadth and/or exclusivity measures across categorical variables (insurance type, network breadth category). Analyses were conducted in R version 4.0.2 (R Project for Statistical Computing).

## Results

Table 1 summarizes the distribution of network breadth across all zip code–network observations. Overall, when viewed from the perspective of US patients, local primary care networks had a mean (SD) breadth of 48.3% (21.8), although one-quarter of network observations had breadth valued at 31.5% or less. Cardiology and hospital networks were slightly larger, with mean (SD) network breadth values of 59.5% (24.9%) and 55.4% (24.7%), respectively. Large-group employer networks had broader coverage than all other network plans (eg, mean [SD] PCP breadth: large-group employer-based plans, 57.3% [20.1]; small-group employer-based plans, 45.7% [21.4]; marketplace, 36,4% [21.2]; MMC, 32.3% [19.3]; MA, 47.4% [18.3]).

**Table 1.** Summary Statistics for Network Breadth Overall and by Insurance Type

Clinical and insurance type	Observations, No.^a^	Zip codes with no physician or hospital within 60-min drive, No. (population in millions)^b^	Percentage of in-network physicians or hospitals within a 60-min drive, %^c^						*P* value^d^
			Mean (SD)	Percentile					
				10th	25th	50th	75th	90th	
Primary care (n = 220 394)^e^	1 248 138	1515 (3.9)	48.3 (21.8)	16.4	31.5	49.5	65.6	77.0	NA
Employer-based									<.001
Large group	511 143	NA	57.3 (20.1)	25.4	45.3	60.4	73.5	80.6	
Small group	318 082	NA	45.7 (21.4)	15.8	28.3	46.7	61.2	75.4	
Marketplace	149 841	NA	36.4 (21.2)	11.4	18.1	33.1	52.2	66.8	
Medicaid managed care	66 370	NA	32.2 (19.3)	9.8	15.2	29.4	46.5	60.7	
Medicare Advantage	202 702	NA	47.4 (18.3)	20.8	35.6	47.8	59.9	71.1	
Cardiology (n = 29 512)^e^	1 173 486	4189 (8.4)	59.5 (24.9)	22.9	40.0	63.3	80.6	89.6	NA
Employer-based									<.001
Large group	482 623	NA	68.5 (22.7)	31.8	57.0	73.4	86.0	91.4	
Small group	297 049	NA	57.1 (24.6)	23.0	36.5	59.2	77.8	89.5	
Marketplace	136 353	NA	45.6 (24.9)	14.9	26.5	42.6	66.6	82.1	
Medicaid managed care	66 016	NA	46.0 (24.6)	13.1	24.1	45.3	66.5	80.6	
Medicare Advantage	191 445	NA	58.7 (21.6)	27.8	43.7	60.5	75.3	85.7	
General acute care hospital (n = 4127)^e^	1 446 413	454 (1.3)	55.4 (25.9)	14.3	36.4	58.6	75.9	87.1	NA
Employer-based									<.001
Large group	621 539	NA	59.5 (24.7)	16.7	45.5	63.6	78.6	87.5	
Small group	355 675	NA	58.8 (24.4)	21.7	41.5	63.2	77.6	87.8	
Marketplace	170 412	NA	51.0 (26.2)	14.1	28.6	50.7	71.4	87.0	
Medicaid managed care	76 382	NA	43.9 (31.9)	7.1	13.3	37.5	73.3	92.0	
Medicare Advantage	222 405	NA	47.3 (25.1)	11.1	26.7	48.4	66.7	81.2	

Abbreviation: NA, not applicable.

^a^ Measures were defined separately for each combination of zip code–specialty or type–plan network. Sample sizes are not equivalent across rows because some areas do not have certain specialties or hospitals within a 60-minute drive.

^b^ As described in the Methods section, a geographic access region was defined separately for each zip code by identifying all physicians and hospitals accessible within a 60-minute drive from the population-weighted centroid. For hospitals, any hospital located outside a 60-minute drive was included if at least 2% of the Medicare inpatient utilization originating from that zip code in 2016 or 2017 was at the hospital.

^c^ Values range from 0% (no area physicians or hospitals in network) to 100% (all area physicians or hospitals in network).

^d^ *P* value based on nonparametric Kruskal-Wallis test for differences in distribution of network breadth across insurance types.

^e^ Number of unique hospitals and physicians.

Network breadth varied across insurance markets (Table 1 and Figure). Large group networks were broader, with 415 549 of 511 143 PCP (81%), 418 793 of 482 623 of cardiology (87%), and 492 225 of 621 539 hospital (79%) zip code–network observations classified as large or extra-large. MA and small-group networks were slightly narrower, with 138 485 of 202 702 MA primary care (68%) and 191 918 of 318 082 small-group primary care (60%) zip code–network observations either large or extra-large. By comparison, just 33% of MMC primary care zip code–network observations fell into the large (14 838 [22%]) or extra large (6942 [11%]) network size category, while nearly half fell into the extra small (7080 [11%]) or small (21 905 [33%]) category. Similarly, 40% of marketplace primary care zip code–network observations were large (37 567 of 149 841 [25%]) or extra-large (22 858 [15%]), and 38% were classified as either extra small (10 773 [7%]) or small (47 041 [31%]).

**Figure.**
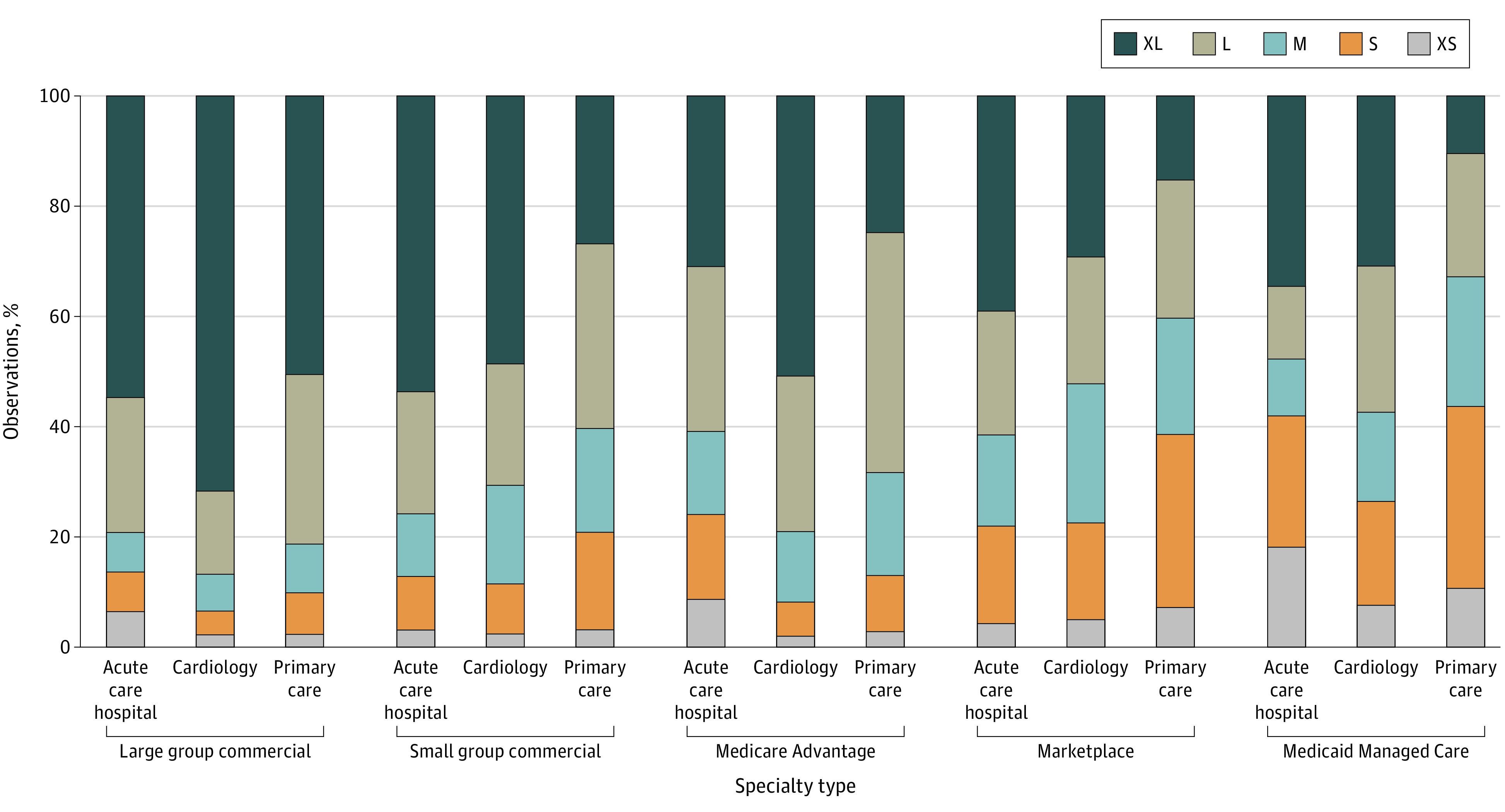
Primary Care Hospital and Physician Network Breadth The figure shows the percentage of observations in each network breadth category by specialty or type and insurance type. Network breadth was defined as the percentage of hospitals or physicians within a 60-minute drive that were in-network for each zip code–specialty type–insurance type–network combination. In total there were 1 248 138 such observations for primary care networks, 1 173 486 for cardiology networks, and 1 446 413 for hospital networks. The continuous network breadth measure was then categorized based on the following sizes: extra small (XS; <10% breadth); small (S; 11%-25%); medium (M; 26%-40%); large (L; 41%-60%); and extra large (XL; >60%).

Measures of exclusivity revealed meaningful differences in the degree of network overlap. Overall, we found that networks had a mean (SD) overlap of 56.5% (11.9) among PCPs (Table 2), while cardiology networks had a mean (SD) overlap of 62.2% (11.9) and hospital networks, 59.6% (12.2). Despite being among the narrowest networks, MMC networks had a larger degree of overlap: mean (SD) overlap was 61.3% (10.5) for primary care, 66.5% (9.8) for cardiology, and 60.5% (12.3) for hospitals (*P* < .001 for all across-market comparisons). By comparison, mean (SD) overlap among large-group networks was 55.0% (11.1) for primary care, 61.0% (12.0) for cardiology, and 58.6% (12.4) for hospitals (*P* < .001 for all comparisons).

**Table 2.** Summary Statistics for Network Exclusivity Overall and by Insurance Market

Clinical and insurance type	Observations, No.^a^	Zip codes with no physician or hospital within 60-min drive, No. (population in millions)^b^	In-network physicians or hospitals within a 60-min drive also in-network in other insurers’ networks, %^c^						*P* value^d^
			Mean (SD)	Percentile					
				10th	25th	50th	75th	90th	
Primary care (n = 220 394)^e^	1 248 138	1515 (3.9)	56.5 (11.9)	43.1	49.7	56.6	64.0	71.0	NA
Employer-based									<.001
Large group	511 143	NA	55.0 (11.1)	41.2	46.8	54.4	62.7	70.4	
Small group	318 082	NA	56.2 (12.0)	44.7	50.4	56.4	62.9	70.3	
Marketplace	149 841	NA	57.4 (14.5)	45.8	53.2	58.5	65.6	72.2	
Medicaid managed care	66 370	NA	61.3 (10.5)	48.5	57.1	62.0	67.6	72.9	
Medicare Advantage	202 702	NA	58.0 (11.1)	44.6	51.5	57.5	65.2	72.3	
Cardiology (n = 29 512)^e^	1 173 486	4189 (8.4)	62.2 (11.9)	48.0	54.5	61.9	70.4	77.3	NA
Employer-based									<.001
Large group	482 623	NA	61.0 (12.0)	45.1	51.7	60.6	69.5	77.3	
Small group	297 049	NA	61.4 (11.7)	48.6	54.1	60.6	69.4	76.2	
Marketplace	136 353	NA	63.2 (13.3)	50.1	56.7	64.0	72.0	77.5	
Medicaid managed care	66 016	NA	66.5 (9.8)	56.0	60.5	66.9	72.8	78.5	
Medicare Advantage	191 445	NA	63.8 (10.6)	51.0	56.5	63.2	71.2	78.1	
General acute care hospital (n = 4127)^e^	1 446 413	454 (1.3)	59.6 (12.2)	45.7	52.4	59.7	66.6	74.2	NA
Employer-based									<.001
Large-group	621 539	NA	58.6 (12.4)	44.1	50.9	58.5	65.8	73.3	
Small-group	355 675	NA	60.2 (10.9)	47.8	54.1	60.6	66.2	73.0	
Marketplace	170 412	NA	60.2 (12.3)	46.9	53.7	60.7	66.8	75.4	
Medicaid managed care	76 382	NA	60.5 (12.3)	47.4	53.0	59.8	67.0	77.1	
Medicare Advantage	222 405	NA	60.4 (13.3)	46.0	52.3	59.8	68.7	76.9	

Abbreviation: NA, not applicable.

^a^ Measures were defined separately for each combination of zip code–specialty or type–plan network. Sample sizes are not equivalent across rows because some areas do not have certain specialties or hospitals within a 60-minute drive.

^b^ As described in the Methods section, a geographic access region was defined separately for each zip code by identifying all physicians and hospitals accessible within a 60-minute drive from the population-weighted centroid. For hospitals, any hospital located outside a 60-minute drive was included if at least 2% of the Medicare inpatient utilization originating from that zip code was at the hospital.

^c^ Values range from 0% (networks are completely exclusive to individual insurance carriers) to 100% (all insurers’ networks include the same in-network physicians/hospitals).

^d^ *P* value based on nonparametric Kruskal-Wallis test for differences in distribution of network breadth across insurance types.

^e^ Number of unique hospitals and physicians.

Table 3 shows state-level variation in network breadth and exclusivity among large-group PCP networks. California had the narrowest (mean [SD] breadth, 42.4% [16.9]) and most exclusive (mean [SD] exclusivity, 47.7% [23.0]) networks, while those in Nebraska were the broadest (mean [SD] breadth, 79.9% [16.6]) and least exclusive (mean [SD] exclusivity, 71.1% [14.6]). In nearly half of states (24 [47.1%]), extra-large networks were the most exclusive. In approximately two-thirds of states, medium (12 states [23.5%]), small (10 [19.6%]) or extra-small (11 [21.6%]) networks were the least exclusive.

**Table 3.** Breadth and Exclusivity of Primary Care Networks Among Large Group Employer-Based Plans by US State

State	Mean (SD) breadth, all networks^a^	Mean (SD) exclusivity^b^						
		All networks	Network breadth category					
			Extra small, <10%	Small, 10%-25%	Medium, 25%-40%	Large, 40%-60%	Extra large, >60%	*P* value^c^^,^^d^
Alabama	63.2 (18.2)	64.4 (9.5)	72.2 (18.2)	65.1 (9.5)	65.6 (18.7)	64.2 (17.7)	64.0 (14.4)	<.001
Alaska	48.0 (17.1)	64.1 (23.3)	65.9 (17.1)	28.7 (18.9)	65.1 (23.3)	68.5 (29.2)	40.7 (21.5)	.003
Arizona	57.9 (16.5)	56.8 (8.7)	44.1 (16.5)	47.6 (9.8)	51.6 (8.7)	57.6 (22.7)	57.7 (19.7)	<.001
Arkansas	65.8 (26.8)	58.2 (31.8)	56.9 (26.8)	54.0 (10.8)	59.0 (31.8)	57.5 (25.1)	58.6 (15.6)	<.001
California	42.4 (16.9)	47.7 (23.0)	49.3 (16.9)	51.2 (6.2)	49.2 (23.0)	46.6 (32.0)	44.7 (43.4)	<.001
Colorado	50.9 (18.0)	56.8 (14.4)	55.0 (18.0)	63.5 (8.6)	60.2 (14.4)	55.8 (31.6)	54.0 (38.7)	<.001
Connecticut	61.4 (22.3)	57.6 (14.2)	48.3 (22.3)	53.3 (12.5)	71.6 (14.2)	68.0 (22.2)	51.5 (39.6)	<.001
Delaware	56.4 (18.6)	49.0 (15.3)	31.5 (18.6)	43.6 (9.8)	55.4 (15.3)	51.1 (13.1)	45.9 (39.8)	<.001
DC	48.5 (20.1)	55.3 (3.1)	73.2 (20.1)	71.4 (10.9)	NA	54.7 (42.5)	46.1 (22.6)	<.001
Florida	51.0 (17.3)	48.4 (21.6)	53.0 (17.3)	60.2 (7.6)	53.8 (21.6)	46.9 (28.6)	46.3 (23.6)	<.001
Georgia	62.9 (12.6)	61.6 (7.6)	59.1 (12.6)	55.9 (7.1)	63.2 (7.6)	64.8 (10.3)	60.2 (10.9)	<.001
Hawaii	68.8 (21.9)	60.4 (31.0)	35.1 (21.9)	58.6 (7.5)	48.5 (31.0)	57.0 (15.6)	61.1 (3.9)	<.001
Idaho	71.0 (21.8)	69.4 (21.5)	72.5 (21.8)	75.4 (12.5)	66.7 (21.5)	68.4 (18.2)	69.3 (20.7)	<.001
Illinois	57.1 (21.0)	49.6 (16.2)	47.6 (21.0)	45.6 (8.3)	55.2 (16.2)	54.9 (27.9)	46.5 (31.3)	<.001
Indiana	61.7 (23.9)	57.1 (26.1)	52.8 (23.9)	55.7 (9.7)	60.2 (26.1)	61.1 (24.7)	56.0 (21.9)	<.001
Iowa	74.2 (23.0)	67.1 (18.2)	53.9 (23.0)	55.3 (12.8)	65.8 (18.2)	73.8 (21.5)	66.9 (21.0)	<.001
Kansas	63.2 (22.8)	62.9 (11.7)	74.4 (22.8)	61.8 (9.6)	65.6 (11.7)	67.7 (26.6)	61.1 (32.4)	<.001
Kentucky	57.6 (23.8)	61.7 (27.7)	63.2 (23.8)	62.0 (8.0)	62.3 (27.7)	68.2 (25.2)	59.3 (21.9)	<.001
Louisiana	65.0 (16.6)	67.6 (13.0)	74.5 (16.6)	69.9 (6.8)	73.8 (13.0)	73.3 (18.3)	64.8 (14.1)	<.001
Maine	60.2 (27.3)	69.4 (18.0)	56.7 (27.3)	66.7 (10.1)	67.5 (18.0)	77.4 (37.3)	68.2 (26.1)	<.001
Maryland	55.8 (18.1)	47.4 (15.3)	50.0 (18.1)	56.8 (8.4)	55.7 (15.3)	49.5 (27.2)	43.8 (26.5)	<.001
Massachusetts	66.0 (17.4)	60.9 (3.7)	63.9 (17.4)	67.6 (9.6)	71 (3.7)	66.7 (7.9)	55.9 (27.6)	<.001
Michigan	60.8 (18.1)	55.0 (20.4)	48.7 (18.1)	54.9 (8.7)	62.7 (20.4)	55.2 (17.6)	54.9 (12.9)	<.001
Minnesota	68.1 (23.2)	56.1 (23.8)	31.2 (23.2)	62.2 (11.7)	56.5 (23.8)	58.1 (13.7)	57.4 (23.4)	<.001
Mississippi	72.6 (15.0)	65.7 (11.2)	57.5 (15.0)	69.9 (8.3)	67.8 (11.2)	67.3 (17.1)	65.6 (7.3)	<.001
Missouri	60.7 (22.0)	61.0 (8.6)	68.7 (22.0)	58.2 (10.2)	67.6 (8.6)	68.7 (24.6)	56.7 (36.1)	<.001
Montana	71.6 (23.2)	70.8 (22.4)	67.5 (23.2)	79.7 (14.2)	68.2 (22.4)	74.9 (21.1)	70.2 (11.4)	<.001
Nebraska	79.9 (16.6)	71.1 (14.6)	72.8 (16.6)	74.2 (8.1)	78.5 (14.6)	76.8 (11.1)	70.8 (14.5)	<.001
Nevada	57.7 (21.8)	51.3 (33.3)	48.3 (21.8)	51.0 (7.5)	54.4 (33.3)	56.8 (9.6)	48.0 (46.6)	<.001
New Hampshire	63.1 (26.8)	60.0 (21.1)	69.5 (26.8)	69 (13.1)	64.6 (21.1)	58.9 (40.1)	58.4 (28.1)	<.001
New Jersey	47.6 (21.0)	43.6 (6.8)	28.1 (21.0)	52.9 (10.0)	47.0 (6.8)	37.4 (45.3)	38.7 (33.0)	<.001
New Mexico	58.8 (21.4)	53.7 (20.2)	39.1 (21.4)	46.1 (12.5)	58.0 (20.2)	55.1 (22.5)	53.5 (37.0)	<.001
New York	52.8 (22.1)	47.1 (14.2)	60.6 (22.1)	50.2 (8.4)	53.7 (14.2)	44.1 (43.0)	45.7 (15.8)	<.001
North Carolina	66.3 (17.1)	64.0 (12.9)	65.7 (17.1)	69.5 (10.0)	70.4 (12.9)	72.9 (11.3)	58.4 (12.0)	<.001
North Dakota	64.8 (27.7)	68.7 (27.9)	72.8 (27.7)	65.4 (13.6)	72.2 (27.9)	74.9 (32.4)	68.0 (21.5)	<.001
Ohio	60.3 (19.8)	57.7 (16.3)	54.9 (19.8)	58.2 (6.9)	63.1 (16.3)	62.9 (19.7)	54.3 (28.3)	<.001
Oklahoma	62.4 (20.6)	58.2 (10.6)	64.0 (20.6)	63.8 (9.6)	55.9 (10.6)	64.4 (25.9)	56.3 (29.8)	<.001
Oregon	60.8 (17.7)	59.2 (10.8)	62.6 (17.7)	61.3 (8.8)	56.0 (10.8)	57.2 (24.7)	60.2 (26.7)	<.001
Pennsylvania	59.6 (18.3)	57.3 (8.4)	56.8 (18.3)	57.5 (11.9)	58.7 (8.4)	58.2 (14.8)	56.6 (31.0)	<.001
Rhode Island	58.6 (22.6)	50.8 (17.7)	55.6 (22.6)	58.0 (8.6)	57.6 (17.7)	58.5 (26.5)	44.9 (35.6)	<.001
South Carolina	65.4 (16.2)	59.7 (15.3)	54.1 (16.2)	51.5 (7.8)	57.8 (15.3)	61.4 (12.9)	59.1 (20.0)	<.001
South Dakota	57.6 (33.1)	58.6 (39.1)	61.7 (33.1)	58.2 (13.2)	66.1 (39.1)	67.2 (36.0)	54.5 (27.0)	<.001
Tennessee	62.6 (21.8)	60.1 (20.9)	56.5 (21.8)	57.8 (9.2)	64.4 (20.9)	63.7 (26.4)	58.5 (18.5)	<.001
Texas	61.4 (20.3)	56.1 (19.0)	58.2 (20.3)	51.8 (7.3)	65.1 (19.0)	58.8 (26.6)	54 (19.7)	<.001
Utah	69.2 (18.9)	64.5 (12.0)	59.1 (18.9)	58.7 (6.9)	67.3 (12.0)	71.4 (24.4)	62.9 (7.9)	<.001
Vermont	67.0 (25.2)	65.3 (19.3)	80.9 (25.2)	78.4 (15.6)	78.1 (19.3)	75.7 (30.6)	61.2 (28.6)	<.001
Virginia	62.9 (17.2)	59.4 (11.5)	60.4 (17.2)	62.3 (11.3)	60.7 (11.5)	60.2 (16.3)	59.0 (20.3)	<.001
Washington	61.2 (18.2)	51.7 (15.8)	69.6 (18.2)	56.5 (10.2)	55.8 (15.8)	52.0 (17.9)	51.1 (18.3)	<.001
West Virginia	55.4 (25.8)	59.1 (32.2)	62.3 (25.8)	60.4 (10.2)	61.3 (32.2)	64.4 (37.9)	55.8 (31.4)	<.001
Wisconsin	59.2 (24.9)	54.3 (27.3)	56.2 (24.9)	53.9 (10.2)	55.8 (27.3)	55.4 (23.8)	53.4 (22.4)	<.001
Wyoming	58.7 (27.1)	64.6 (30.2)	75.7 (27.1)	54.0 (17.9)	64.2 (30.2)	72.1 (25.9)	61.5 (37.1)	.17

^a^ Values range from 0% (no area physicians or hospitals in network) to 100% (all area physicians and hospitals in network).

^b^ Values range from 0% (networks are completely exclusive to individual insurance carriers) to 100% (all insurers’ networks include the same in-network physicians and hospitals).

^c^ *P* value based on nonparametric Kruskal-Wallis test for differences in distribution of network breadth across markets.

^d^ *P* value for test of differences in exclusivity across network breadth categories was *P* < .001 for every state except Wyoming (*P* = .17).

The size and exclusivity of networks also varied across insurer and market concentration levels (Table 4). Among large-group commercial plan networks, the broadest (median [IQR], 75.0% [60.0%-83.1%]) and least exclusive (median [IQR], 63.7% [52.4%-73.7%]) primary care networks were observed in markets with concentrated primary care and insurance markets. By comparison, the narrowest (median [IQR], 54.6% [46.8%-67.6%]) and most exclusive (median [IQR], 49.4% [41.9%-56.9%]) networks were observed in markets with the least market concentration among both clinicians and insurers.

**Table 4.** Breadth and Exclusivity of Large Group Networks by Insurance and Market Structure Type

Physician and hospital market concentration type (% of US population in category)	Median (IQR) network breadth (% of US population in category)^a^			Median (IQR) network exclusivity (% of US population in category)^b^		
	Not concentrated (9%)^c^	Moderately concentrated (62%)^c^	Concentrated (28%)^c^	Not concentrated (9%)^c^	Moderately concentrated (62%)^c^	Concentrated (28%)^c^
**Primary care (n = 220 394)**						
Not concentrated (88%)^c^	54.6 (46.8-67.6)	58.9 (44.6-72.6)	62.4 (43.1-73.4)	49.4 (41.9-56.9)	53.9 (46.9-61.6)	54.4 (46.2-62.7)
Moderately concentrated (9%)^c^	65.6 (52.6-77.2)	70.6 (51.1-80.2)	71.9 (54.4-80.1)	60.7 (52-64.4)	61.4 (53.9-68.7)	63.6 (56.0-71.8)
Concentrated (3%)^c^	NA^d^	71.4 (55.6-81)	75.0 (60.0-83.1)	NA^d^	64.1 (53.5-71.3)	63.7 (52.4-73.7)
**Cardiology (n = 29 512)**						
Not concentrated (53%)^c^	70.8 (66.5-80.3)	68.5 (51.4-82.4)	65.0 (48.8-82.8)	54.5 (47.5-59.5)	55.9 (48.1-63.7)	55.5 (49.9-59.9)
Moderately concentrated (22%)^c^	76.8 (52.1-81.3)	81.2 (64.2-89.1)	81.2 (69.7-90.0)	62.9 (59.1-64.8)	65.4 (58.8-74.3)	70.0 (62.4-74.8)
Concentrated (25%)^c^	80.6 (62.5-88.8)	81.5 (66.0-90.0)	83.3 (66.7-91.5)	69.2 (59.8-75.0)	69.1 (60.9-75.6)	70.9 (61.7-78.1)
**General acute care hospital (n = 4127)**						
Not concentrated (1%)^c^	NA^d^	NA^d^	NA^d^	NA^d^	NA^d^	NA^d^
Moderately concentrated (74%)^c^	64.0 (52.0-80.0)	66.7 (48.0-80.0)	58.3 (41.7-75)	59.3 (52.3-68.3)	58.1 (50.0-64.4)	57.6 (51.5-65.2)
Concentrated (24%)^c^	70.0 (37.5-83.3)	64.7 (42.9-78.9)	60.7 (42.9-75.0)	58.7 (52.6-66.7)	60.6 (52.7-68.4)	59.3 (50.0-68.4)

Abbreviation: NA, not applicable.

^a^ Values range from 0% (no area physicians or hospitals in network) to 100% (all area physicians and hospitals in network).

^b^ Values range from 0% (networks are completely exclusive to insurers) to 100% (all insurers’ networks include the same in-network physicians and hospitals).

^c^ Based on Hirschman-Herfindahl Index values of 0 to 1500 (not concentrated); 1500- to 2500 (moderately concentrated); and 2500 and 10 000 (concentrated).

^d^ Data not shown; <1% total population in cell, or market combination does not exist in data.

## Discussion

This study found variation in the breadth and exclusivity of physician and hospital networks. Networks were broader in employer-sponsored (large-group) plans and narrower in individually purchased (marketplace) and MMC plans. Despite being narrower, Medicaid networks were more connected with other networks in their area. Breadth and exclusivity also did not go hand-in-hand: in many states, the broadest networks had a lower degree of overlap with other networks in the same area. Finally, areas characterized by high insurer and (especially) physician and hospital market concentration had broader and less exclusive networks.

As the first study that we are aware of to examine networks for all major insurance types, this research highlights how the structure of plan networks can serve as an important determinant of care affordability and continuity in the United States. Our previous research has documented frequent churn that occurs as people change insurance plans.^27,28,29^ In insurance markets characterized by churn, a concern is that individuals changing insurance plans must make difficult choices regarding affording and maintaining preferred clinical relationships on the one hand and establishing new in-network relationships on the other. This concern is motivated by research documenting noteworthy disruptions in care and an increased reliance on emergency departments associated with changes in insurance plans.^13,14,15,16^ Narrow network plans have also been shown to have lower premiums,^30^ yet little is known about whether selection of a less expensive, narrow network plan increases the likelihood that patients would need to find new clinicians once they enroll. Finally, the patient financial protections established by the Patient Protection and Affordable Care Act—such as annual limits on patient out-of-pocket spending and prohibitions on annual and lifetime insurer spending maximums—apply to nearly all private health insurance plans but only for care received by in-network clinicians and hospitals. Given these considerations, it is important to know whether patients will be able to affordably maintain preferred clinical relationships if and when they change insurance plans.

Our findings demonstrate that health care networks can be narrow but still exhibit sizeable overlap with other area networks. Indeed, in many states, smaller networks exhibited the most overlap across insurers’ networks. While this finding may seem counterintuitive, it is consistent with the observation that large networks can also be exclusive (eg, 75% of local physicians could contract exclusively with a single insurer, while the remaining 25% contract with a variety of different insurers).

Our results by state indicate that California had the narrowest and most exclusive networks. This is not surprising given that California is also home to Kaiser Permanente—perhaps the most well-known example of an exclusive, clinically integrated large insurer. But it is important to emphasize that a highly exclusive network is not necessarily a clinically integrated network. Indeed, we found that the most exclusive networks were in the most competitive market environments. As insurers compete with each other and negotiate with physician and hospital groups over payment rates and inclusion in their networks, clinicians could find themselves in exclusive networks with other physicians and facilities that do not share the same health information technology and clinical guidelines. These dynamics could also affect the quality of care if referrals within an exclusive network are restricted to unfamiliar or nonpreferred specialists and facilities. Further research is needed to investigate how our measures of breadth and exclusivity are associated with measures of clinical integration, referral patterns, and health care quality.

### Limitations

A well-known limitation of health care network research is inaccuracies in directory data. Our study put in place several safeguards to reduce errors, but nevertheless, some limitations remain. First, Vericred has internal quality assurance (QA) process to work directly with carriers to limit errors. This QA process ensures that Vericred’s commercial clients—which include major human resources management firms, health plans, and health insurance shopping websites—have access to high-quality, up-to-date network data. Second, in addition to regular audits, insurers now face stronger regulatory oversight: as of early 2016, marketplace and MA carriers face steep fines for having out-of-date and inaccurate directories. Third, according to CMS audits of MA networks, the most common reason for errors is incorrect information on clinic location and contact information for in-network clinicians (74% of all errors). A frequent reason for the other 26% of errors (eg, physician should not be listed as in-network) is retirement and moving from the area or clinic/facility. Our validation approach using biannual (IQVIA) and annual (AHA and Physician Compare) data should reduce these errors as a source of bias in our analyses because these data sources rely on frequent canvassing and PECOS data to ensure up-to-date information on location, active status, and specialty. These validation exercises go beyond the safeguards for data accuracy already in place at Vericred and among insurance carriers and result in total active physician counts for our study that align well with counts from other external data sources (eg, the American Medical Association Masterfile) (eAppendix in the Supplement).

## Conclusions

To our knowledge, this study provided the first national snapshot of the size and exclusivity of insurance networks across all markets. These findings demonstrate that the size and connectedness of health care networks can be important determinants of care affordability and continuity in the United States.

## Notes

Supplement. **eAppendix. **Supplementary Methods and Analyses
