# Supplementary material for: Breadth and Exclusivity of Hospital and Physician Networks in US Insurance Markets
Source: JAMA Netw Open. 2020 Dec 17;3(12):e2029419. doi: 10.1001/jamanetworkopen.2020.29419 (PMC7747020; doi:10.1001/jamanetworkopen.2020.29419)
Supplement: Supplement. — eAppendix. Supplementary Methods and Analyses [file jamanetwopen-e2029419-s001.pdf]

Supplemental Online Content

Graves JA, Nshuti L, Everson J, et al. Breadth and exclusivity of hospital and physician networks in US insurance markets. *JAMA Netw Open*. 2020;3(12):e2029419. doi:10.1001/jamanetworkopen.2020.29419

eAppendix. Supplementary Methods and Analyses

This supplemental material has been provided by the authors to give readers additional information about their work.

© 2020 Graves JA et al. *JAMA Network Open.*

eAppendix. Supplementary Methods and Analyses

Table of Contents

Introduction 3

Geographic Measures 3

- Defining Geographic Accessibility 3
- Isochrones for an Example ZIP Code 3
- Validation of Hospital and Physician Data 4
- Network Landscape Files 9

Measures of Market Concentration 12

- Physician HHI 13
- Hospital HHI 13
- Insurer HHI 13

Network Measures 14

- Network Definitions 14
- Example Network 14

Supplemental Results 22

© 2020 Graves JA et al. *JAMA Network Open.*

Introduction

This document provides methodological details and supplemental analyses for the manuscript. This report was compiled on 2020-10-16 using R version 4.0.2 (2020-06-22). Replication code is available on github.

Geographic Measures

Defining Geographic Accessibility

Our primary measure of geographic accessiblity was based on a driving-time based isochrone centered on the population-weighted centroid of ZIP-code tabulation areas (ZCTAs). We identified centroids based on 2010 Census block population data using the Geographic Correspondance Engine at the Missouri Census Data Center. We constructed thirty- and sixty-minute isochrones for each ZIP using the Mapbox Application Protocol Interface (API).

Isochrones for an Example ZIP Code

eExhibits 1 and 2 map the isochrones for ZIP 53005 (blue polygon) near Milwaukee, WI. Also plotted in eExhibit 2 is the ZIP's surrounding county (solid black line) and the locations of primary care physicians (blue dots) and hospitals (pink squares) within the 30-minute isochrone.

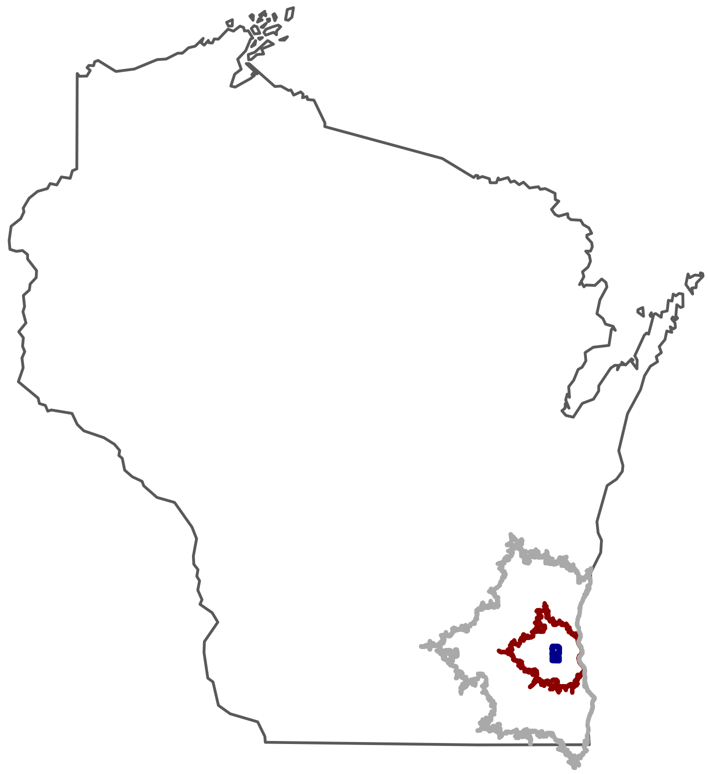

eExhibit 1: 30- and 60-Minute Isochrones for Example ZIP

© 2020 Graves JA et al. *JAMA Network Open.*

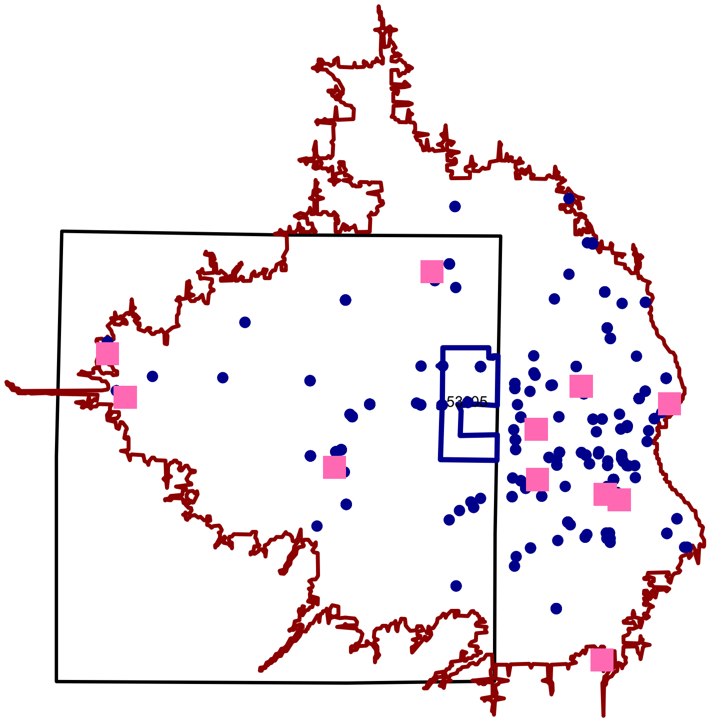

eExhibit 2: 30-Minute Isochrone and Surrounding County Boundary Points for Example ZIP, with Geographic Location of Primary Care Physicians (circles) and Hospitals (squares)

As eExhibit 2 shows, isochrone-based definitions of geographic accessibility do not (arbitrarily) limit measures of access to only those providers located within geo-political boundaries (e.g., the county). Indeed, eExhibit 1 demonstrates (for the 60-minute isochrone) that geographic access can often reach into neighboring states.

For our primary results we utilize a 60-minute isochrone for every ZIP code, however in sensitivity analyses we considered a 30-minute isochrone for ZIPs located within metropolitan core-based statistical areas (i.e., non-rural areas).

Validation of Hospital and Physician Data

While the Vericred data included the specialty (for physicians) and geocoded address location for each clinician and hospital facility, we included additional sample inclusion safeguards to ensure that the "denominator" of geographically accessible clinicians/facilities from a given ZIP code included only those currently practicing in the area.

Common Reasons for Network Errors

According to **CMS audits**, the most common reason for provider network errors is incorrect information on clinic location and contact information for in-network clinicians (74% of all errors; see table below). A frequent reason for the other 26% of errors (e.g., physician should not be listed as in-network) is retirement and moving from the area or clinic/facility. This often occurs because

insurance carriers have historically been fairly passive about updating provider network data (i.e., they do not routinely canvass the directory to ensure that every provider is still practicing at each location).

Provider Directory Deficiency Types by Occurance Based on CMS Audit of Medicare Advantage Networks

	Percent of Errors
Provider should not be listed in the directory at this location	39.61
Provider should not be listed at any of the directory-indicated locations	26.43
Phone number needs to be updated	13.09
Address needs to be updated	6.91
Address (suite number) needs to be updated	4.53
Provider IS accepting new patients	4.46
Provider is NOT accepting new patients	4.19
Specialty needs to be updated	0.46
Provider name needs to be updated	0.32

To address this limitation we augmented the insurance network data with a comprehensive validation process to ensure that the information we used reflected the best possible information on the specialty and active status of physicians and hospitals in the U.S. We describe our validation process separately for hospitals and physicians below.

Hospital Data Validation Process

One challenge with hosptial network data is that often, only a single national provider identifer (NPI) is provided for facilities in a given network. However, hospitals often have multiple NPIs registered for different buildings and units. For example, our final sample includes 4,127 unique facilities that, collectively, have 14,680 NPIs associated with them. This creates opportunities for data errors because the NPI number listed by one carrier may not be the same NPI provided by another. Without further adjustment we might incorrectly determine that the same facility does not appear in both insurers' networks, which would invalidate our measures of exclusivity.

We addressed this challenge by constructing a master hospital NPI crosswalk that identified all NPIs associated with a hospital. This crosswalk was constructed using American Hospital Association (AHA) and National Plan and Provider Enumeration System (NPPES) data. Following the methodology outlined by Cooper et al (Quart J. Economics 2019), the following steps were used to construct the crosswalk:

1. Compile all variations of AHA ID/hospital name/address/city/state/ZIP Code in the 2015 AHA survey data.
2. Add NPI from the AHA survey files, beginning with the most recent year.
3. Make sure there is only one NPI per AHA ID. If more than one AHA ID have the same NPI, look up in the CMS NPI Registry to resolve the discrepancy.

© 2020 Graves JA et al. *JAMA Network Open.*

4. Check all NPIs in the CMS NPI Registry to make sure they are valid and accurate. Remove invalid NPIs.
5. Look up hospitals in the NPI Registry that do not have a NPI in AHA by name and address. Attach NPI to the AHA file when a match is found.
6. Extract all organizational rows from the CMS NPI Registry where primary taxonomy code is for a hospital (287300000X, 281P00000X, 281PC2000X, 282N00000X, 282NC2000X, 282NC0060X, 282NR1301X, 282NW0100X, 282E00000X, 286500000X, 2865C1500X, 2865M2000X, 2865X1600X, 283Q00000X, 283X00000X, 283X00000X, 283XC2000X, 282J00000X, 284300000X) or hospital unit (273100000X, 275N00000X, 273R00000X, 273Y00000X, 276400000X).
7. Match AHA compiled address file to the hospital NPI file on NPI. Add AHA number to the hospital NPI file and mark the NPI as 'PRIMARY' NPI for that hospital.
8. Match remaining rows in the hospital NPI file according to the following hierarchy:
    1. Organization name, address1, city, state, ZIP Code
    2. Address1, city, state, ZIP Code, similar organization name
    3. Other organization name, address1, city, state, ZIP Code
    4. Address1, city, state, ZIP Code, similar other organization name
    5. Address, city, state, ZIP Code, different name (validated name changes via web search)1
    6. Organization name, similar address1, city, state, ZIP Code2
    7. Other organization name, similar address1, city, state, ZIP Code
    8. Similar organization name, similar address1, city, state, ZIP Code
    9. Similar other organization name, similar address1, state, ZIP Code
    10. Medicare number, city, state, ZIP Code
9. When a match is found, append AHA ID and 'PRIMARY' NPI.
10. Some hospitals in the NPI Registry were not in the AHA survey data files. For these hospitals, we pick one NPI as 'PRIMARY' and, using the match steps outlined above, add an 'X' to the AHA ID column and append the 'PRIMARY' NPI to all matched rows.

Physician Data Validation Process

To validate physician specialty, active status, and practice location information we drew on 2019 data from IQVIA and Hospital Compare. IQVIA routinely canvasses office-based physician clinics nationwide to collect information on specialty and organizational relationships, among other things. Because the IQVIA data are primarily sold for marketing purposes, the data contain up-to-date contact information (including clinic ZIP code) for nearly all active office-based (as well as some facility-based) physicians nationwide.

One downside to the IQVIA data is that the canvassing frame (mostly office-based physician clinics) undercounts certain physician specialties and types. The Medicare Payment Advisory Commission, for example, has found that IQVIA data do not cover roughly 30 percent of physicians who bill Medicare in a given year. Not surprisingly given its sampling frame, the IQVIA undercount is

concentrated among certain hospital-based specialties (e.g., radiologists, pathologists, and anesthesiologists) and among physicians working predominantly in hospitals and other facilities (e.g. emegency medicine physicians and general internists working as hospitalists and intensivists).

To address these data gaps and to further validate the information contained in the IQVIA and Vericred data we drew on additional December 2019 data from Physician Compare. Physician Compare is updated twice monthly by CMS and captures current information on clinic addresses, primary specialty, and licensure data. Critically, Physician Compare captures all active physicians who submitted a Medicare claim within the last 12 months of data collection, or who newly registered within the Medicare Provider Enrollment, Chain, and Ownership System (PECOS) within 6 months of data collection. Thus, it is effectively a continually-updated census of physicians billing Medicare. That said, Physician Compare also has its own limitations since it will not capture physicians who do not bill Medicare.

We geocoded the location of all physicians using address data from these three data sources (Vericred, IQVIA and Physician Compare) based on the following process. Generally speaking, this process utilized the most detailed address information available from the data sources with the broadest agreement on clinic/facility location. *Our process also reflects the assumption that any address information in IQVIA and Hospital Compare was more current than information in the Vericred data.* This assumption rests on the observation (relayed to us by Vericred) that their address data was largely based on the NPPES (which often list a home address if the physician has licensure data sent there, and is less frequently updated to reflect changes in address, residency/training status, and clinic location) and network scrapes from the web. Likewise, we assumed that if a physician did not appear as active in either IQVIA or Hopstial Compare data, then he or she was likely not directly engaged in patient care in 2019. Finally, our process ensured that we identified all clinics where a physician practices.

1. We first compared unique NPIs in either IQVIA and Physician Compare to get a "master" listing of active physicians in the U.S.
2. We then took all NPIs, their specialty, and the geographic location of all listed clinics from this master listing to the Vericred data.
3. We next determined whether the clinic ZIP code listed in the Vericred data matched any of the ZIP codes associated with the NPI in the IQVIA and Hospital Compare data.
4. We recorded which ZIPs agreed across the various data sources (e.g., ZIP codes agree in Vericred, IQVIA and Hospital Compare; ZIP codes agree in Vericred and IQVIA; ZIP codes agree in Vericred and Hospital Compare; ZIP codes agree in IQVIA and Hospital Compare)
5. We then used the following hierarchy to assign the most comprehensive address information to each NPI, based on the observation that both Vericred and Hospital Compare have clinic addresses, while IQVIA only lists the clinic ZIP code.
    1. If all three agree, use all addresses from Hopsital Compare. We use Hospital Compare addresses and not Vericred addresses because Hospital Compare is more routinely updated based on Medicare billing, while Vericred data may lag depending on individual carriers' update schedules [n=152,166 (18%)].

© 2020 Graves JA et al. *JAMA Network Open.*

2. If Vericred agress with Physician Compare, use address locations from Physician Compare [n = 204,836 (24%)].
3. If Vericred agrees with IQVIA, use address location from Vericred [n = 25,413 (3%)].
4. If IQVIA and Physician Compare agree, but neither agrees with Vericred, use clinic location information from Physician Compare [n = 149,171 (18%)].
5. If IQVIA does not include the NPI, but Physician Compare and Vericred disagree on location, use clinic location information from Physician Compare [n = 223,821 (26%)].
6. If Physician Compare does not include NPI, but IQVIA and Vericred disagree on location, use ZIP centroid from all clinic locations in IQVIA [n=91,162 (11%)].

6. With this master data created, we then geocode each address (or population-weighted ZIP centroid, in cases where IQVIA data were used). These geocoded coordinates are then used to isolate the physicians within each ZIP code's isochrone.

To ensure consistency, we utilized primary specialty information from the data source used to inform the clinic address locations. For example, if Hospital Compare was used to determine the address or addresses for a given NPI, then we utilized the primary specialty information from Hospital Compare for that NPI. Generally speaking the data sources agreed on specialty, though there was some disagreement between IQVIA and Hospital Compare for certain sub-specialties. For example, among physicians identified as emergency medicine in either IQVIA or Hospital Compare, 8% had a different primary specialty in one of the data sources. Likewise, there was 6.9% disagreement for Cardiology. All other subspecialties considered had much lower rates of disagreement (e.g., 0.3% for anesthesiology, 0.7% for radiology, 0.3% for behavioral health, 0.9% for othopedic surgery, and 3.6% for primary care).

			Specialty and Location Data Source			
	Unique Count	Total Observations[1]	AHA	Physician Compare	IQVIA	Vericred/NPPES
Acute Care Hospital	4,127	14,680	14,680			
Physician Specialties						
Cardiology	29,483	80,152		71,026	6,737	2,389
Primary Care	220,001	365,215		315,320	40,002	9,893

[1] Includes observation for each clinic location.

eExhibit 3: Total and Unique Counts of Hospitals and Physicians by Input Data Source

Based on this process we then compared counts of active physicians by specialty to another data source: the 2015 American Medical Association Master File. While these comparisons were somehat applies-to-oranges–for example, the AMA data were from 2015 and our data reflected 2019 counts, and the AMA data did not include clinical psychologists in its definition of behavioral

health physicians–our counts aligned well with counts of active physicians in the masterfile.

	2019 Analytic Sample	2015 AMA Master File
Anesthesiology	44,750	41,306
Behavioral Health	63,997	37,717[1]
Cardiology	29,512	27,803
Emergency Medicine	42,456	39,547
Orthopedic Surgery	27,245	19,142
Primary Care	220,394	224,998
Radiology	35,549	27,505

[1] Master file specialties include psychiatrists only, and excludes clinical psychologists captured in the SK&A and Hospital Compare data
Source: https://www.aamc.org/data-reports/workforce/interactive-data/active-physicians-sex-and-specialty-2015

eExhibit 4: Total Counts of Physicians by Specialty and Data Source

Network Landscape Files

Our analyses also requried identification of the set of plan networks available in each ZIP. For some markets (marketplace, small-group) this was straighforward beacuse CMS and Vericred had crosswalks that allowed us to map each plan marketed in the ZIP to its network. However, for other markets (Medicaid managed care, large group, and Medicare Advantage) we had to infer the set of networks using additional data sources. Below, we describe the process of identifying provider networks available and/or marketed in each ZIP.

Marketplace and Small Group Plans

To identify marketplace and small group plans available in a ZIP code, we first mapped each (population-weighted) ZIP centroid to its county and 3-digit ZIP code. States, **in conjunction with CMS**, define health insurance rating areas for marketplace and small group plans based on clusters of contiguous counties or 3-digit ZIP codes. This mapping allowed us to map each ZIP code to its geographic rating area.

Crosswalks provided by CMS and Vericred (including **HIX Compare**, which Vericred curates) facilitated matching of each rating area to the set of marketplace and small group plans marketed in the area. We then mapped each of these plans to its network using Vericred crosswalks. The resulting output provided us with the set of networks available in the ZIP.

Based on this process, of all possible ZIP-network matches, we had network data for 99.72% for marketplace and 97.7% for small-group markets.

Medicaid Managed Care Plans

Identifying the set of available Medicaid managed care plans required the use of enrollment data for individual insurance carriers. These data were obtained for January 2019 from Decision Resources Group (DRG). Specifically, the DRG data contained county-level enrollment (based on enrollee

© 2020 Graves JA et al. *JAMA Network Open.*

residence and listed separately by insurance carrier and market type) submitted by health plans as part of DRG's National Proprietary Census.

For each county we identified the set of carriers with non-zero Medicaid managed care enrollment based on beneficiary residence. We then mapped each ZIP's population-weighted centroid to the underlying county to match this set of carriers to every ZIP code in the county. We then matched this set of carriers to the Vericred data to identify the Medicaid networks available.

Notably, certain states (AL, AK, AR, CT, ID, ME, MT, NC, OK, SD, VT) do not utilize Medicaid managed care and consequently were excluded from our analyses of Medicaid networks. In addition, Vericred did not capture network information for all Medicaid carriers nationwide. However, our data matching process determined that the Vericred data captured networks for 76.8% of Medicaid managed care enrollment nationwide. Table below shows, however, that the fraction of matched networks varied across states.

© 2020 Graves JA et al. *JAMA Network Open.*

	Percent MMC Enrollment		Percent MMC Enrollment
US	76.8	MS	100.0
AK[1]	0.0	MT[1]	0.0
AL[1]	0.0	NC[1]	0.0
AR[1]	0.0	ND[1]	0.0
AZ	64.2	NE	66.7
CA	87.3	NH	46.2
CO	47.4	NJ	85.8
CT[1]	0.0	NM[1]	90.0
DC	76.7	NV	0.0
DE	100.0	NY	95.4
FL	89.2	OH	98.9
GA[1]	73.9	OK[1]	0.0
HI	86.1	OR	34.1
IA	68.1	PA	88.5
ID[1]	0.0	RI[1]	3.6
IL	68.7	SC	90.3
IN[1]	59.4	SD[1]	0.0
KS	68.0	TN	72.3
KY	60.4	TX	77.6
LA	75.2	UT	100.0
MA	39.2	VA	51.9
MD[1]	69.9	VT[1]	0.0
ME[1]	0.0	WA[1]	76.8
MI	55.1	WI	74.6
MN	93.6	WV	70.0
MO	100.0	WY	0.0

[1] State does not contract with Medicaid Managed Care organizations.

eExhibit 5: Percent of 2019 Medicaid Managed Care Enrollment Captured in Vericred Network Data, Overall and by State

© 2020 Graves JA et al. *JAMA Network Open.*

Commercial (Large-Group) Networks

We identified the set of commercial (large-group self insured) carriers using a similar method as for Medicaid managed care. Specifically, we used the county-level DRG data to isolate the set of carriers with non-zero enrollment (based on beneficiary residence). We then mapped this set of carriers to each ZIP code with a population-weighted centroid in the county, as well as to the set of networks associated with those carriers in the Vericred data. The Vericred data also included a market identifier for each network, allowing us to identify only those large-group networks associated with carriers in the ZIP.

Because the DRG data included enrollment data on all carriers in the ZIP we were able to estimate, to a rough approximation, what fraction of carriers matched between the DRG data and the Vericred large group networks. Based on the above process we matched networks to carriers with approximately 64% of large-group self insured enrollment nationwide.

One challenge to identifying large group networks is that we only knew enrollment at the carrier level, not the plan level. Moreover, even if we knew plan-level enrollment figures for each ZIP, we lacked a crosswalk mapping each network to each plan. Therefore, our analysis of large group newtorks has several limitations: (1) we were not able to capture large-group networks for carriers covering 36% of enrollment; (2) we could not verify that, among the 64% for whom we did have networks, those networks were exhaustive of the networks offered by the carriers. In other words, unlike all other markets considered, while we might match at the carrier level, we lacked data to verify that we matched at the network level.

Medicare Advantage

To isolate the MA plans available in the area we used the July 2019 enrollment by contract/plan/state/county files constructed by CMS. We first mapped each ZIP centroid to its county, then matched this county information with MA enrollment data in the county. Because some individuals live in multiple locations and thus may buy their MA plans from a different residence, we restricted the set of MA networks to only those with at least 2% enrollment market share in the county. We then used a crosswalk mapping each MA plan ID to its network ID in the Vericred data using a crosswalk provided to us by Vericred. This process resulted in matching networks to plans covering 97% of Medicare Advantage enrollment in July 2019.

Measures of Market Concentration

Our analysis relies on measures of market concentration for hosptials, physicians and insurers based on the Hirschman-Herfindahl index, or HHI. HHI measures are commonly used to quantify the degree of concentration among market participants, and are constructed based on the sum of squared market shares (expressed as a percentage) within a defined market. Values closer to zero indicate markets in which market shares are more evenly distributed among multiple market participants. By comparison, an HHI value of 10,000 (i.e., 100^2) indicates a completely consolidated market.

The geographic market definition we used for all HHI measures was the commuting zone. Commuting zones are comprised of geographically contiguous counties with strong within-area clustering of commuting ties between residental and work county, and weak across-area ties. Thus, this set of approximately 600 commuting zones nationwide approximates areas of economic activity over which it is reasonable to consider measures of market concentration. We used commuting zones based on Census data from 2010 constructed by researchers at **Penn State**.

While we provide basic details on our HHI measures here, we provide extensive documentation, code, and analyses of HHI methods in our github document **Defining Markets for Health Care Services**.

Physician HHI

Our measure of physician HHI relies on the methodology outlined in Richards et al (*Health Serv Res* 2017). As described in that study:

> [HHI measures reflect] the allocation and organization of all physician specialists within a given geographic area. In other words, we capture if an insurer would have relatively few or many physician practices to negotiate with in regard to enrollee access and payments (as well as other contractual terms).

To construct our HHI measure by commuting zone we utilized detailed information on organizational relationships reported in the IQVIA data. By assigning each physician/NPI to her organization, we were able to construct count measures of the total number of physicians per organization in an area. These count measures were the basis for the market shares that fed into standard HHI equations (i.e., the sum of squared market shares).

Hospital HHI

Our measures of hosptial HHI draw on the 2016-2017 CMS hospital service area files that capture ZIP-level utilization of each general acute care hosptial. We use these data to identify the set of hospitals that treat Medicare patients who reside in each ZIP. We calculate an HHI value for each ZIP code and then aggregate up to the commuting zone level by taking a weighted (by 2010 Census population) average across ZIPs with centroids located within the commutizing zone.

Insurer HHI

To construct measures of insurer HHI we calculated total enrollment by insurance carrier in each county, then aggregated these enrollment totals up to the commuting zone level. These market shares then fed through standard HHI formulas. We did not calculate HHIs separately by market (e.g., large group, small group, etc.) under the assumption that insurers use the full weight of their enrollment totals as leverage when negotiating networks.

© 2020 Graves JA et al. *JAMA Network Open.*

Network Measures

Network Definitions

We measure provider network connections using binary bipartite networks that capture in-network relationships between the (N_{gs}) individual hospitals or physicians of a given specialty (s) and the (K_g) provider networks available in a geographic market (g). That is, these $N_{gs} \times K_g$ networks capture binary information on whether each physician/hospital is in-network for each provider network available in the geographic market. As described above, the N_{gs} rows of these matrices are determined by the total number of physicains/hospitals within the isochrone, while the K_g columns of the matrix are determined by the number of networks marketed as part of plans available in the ZIP. Thus, each combination of ZIP and specialty type receives its own bipartite matrix.

Physicians and hospitals are identified using their national provider identifier (NPI). In the case of hospitals (which, as noted above, can have multiple NPIs), we utilize a single NPI so we do not over-count hosptials in the bipartite networks.

We further define another set of binary matrices to identify networks offered by the same insurer, and networks for different insurance markets (large-group, small-group, Medicaid managed care, Medicare Advantage, and marketplace). These matrices are utilized to construct measures separately by market, and to construct measures of the degree of connections across insurers.

Example Network

eExhibit 6 below provides an example bipartite network matrix that will be used throughout this section. In this example, there are 10 clincians (NPIA-NPIJ), 10 provider networks (A1-G4), and 7 insurers (A-G). One insurer (G) offers 4 separate networks (G1, G2, G3, and G4). Each cell contains binary information on whether the clinician is in-network the network.

© 2020 Graves JA et al. *JAMA Network Open.*

Clinician ID	Network ID (Insurer-Network)									
	A1	B1	C1	D1	E1	F1	G1	G2	G3	G4
NPIA	1	1	0	0	0	0	0	0	0	0
NPIB	1	1	1	0	0	0	0	0	0	0
NPIC	0	1	1	1	1	1	0	0	0	0
NPID	0	0	1	1	1	1	0	0	0	0
NPIE	0	0	1	1	1	1	0	0	0	0
NPIF	0	0	1	1	1	1	1	0	0	0
NPIG	0	0	0	0	0	1	1	1	1	0
NPIH	0	0	0	0	0	0	1	1	1	1
NPII	0	0	0	0	0	0	1	1	1	1
NPIJ	0	0	0	0	0	0	0	1	1	1

eExhibit 6: Example Bipartite Matrix

The bipartite matrix can also be converted into a **unipartite** matrix that summarizes the total number of connections among the 10 provider networks:

$$UP = BP'BP$$

The resulting unipartite matrix is depicted in eExhibit 7 .

© 2020 Graves JA et al. *JAMA Network Open.*

Network ID	Network ID									
	A1	B1	C1	D1	E1	F1	G1	G2	G3	G4
A1	2	2	1	0	0	0	0	0	0	0
B1	2	3	2	1	1	1	0	0	0	0
C1	1	2	5	4	4	4	1	0	0	0
D1	0	1	4	4	4	4	1	0	0	0
E1	0	1	4	4	4	4	1	0	0	0
F1	0	1	4	4	4	5	2	1	1	0
G1	0	0	1	1	1	2	4	3	3	2
G2	0	0	0	0	0	1	3	4	4	3
G3	0	0	0	0	0	1	3	4	4	3
G4	0	0	0	0	0	0	2	3	3	3

eExhibit 7: Example Unipartite Matrix Summarizing the Number of Shared Connections

The unipartite matrix can also be plotted to visualize the degree of connections among insurers. In eExhibit 8 below, each network is represented as a node. The color of the nodes differentiates among the 7 insurers in the example. The width of the edge lines connecting the nodes are proportional to the number of connections: the wider the line, the more “shared” providers the networks have. The total number of connections (i.e., the values in the cells in eExhibit 7) is also shown.

© 2020 Graves JA et al. *JAMA Network Open.*

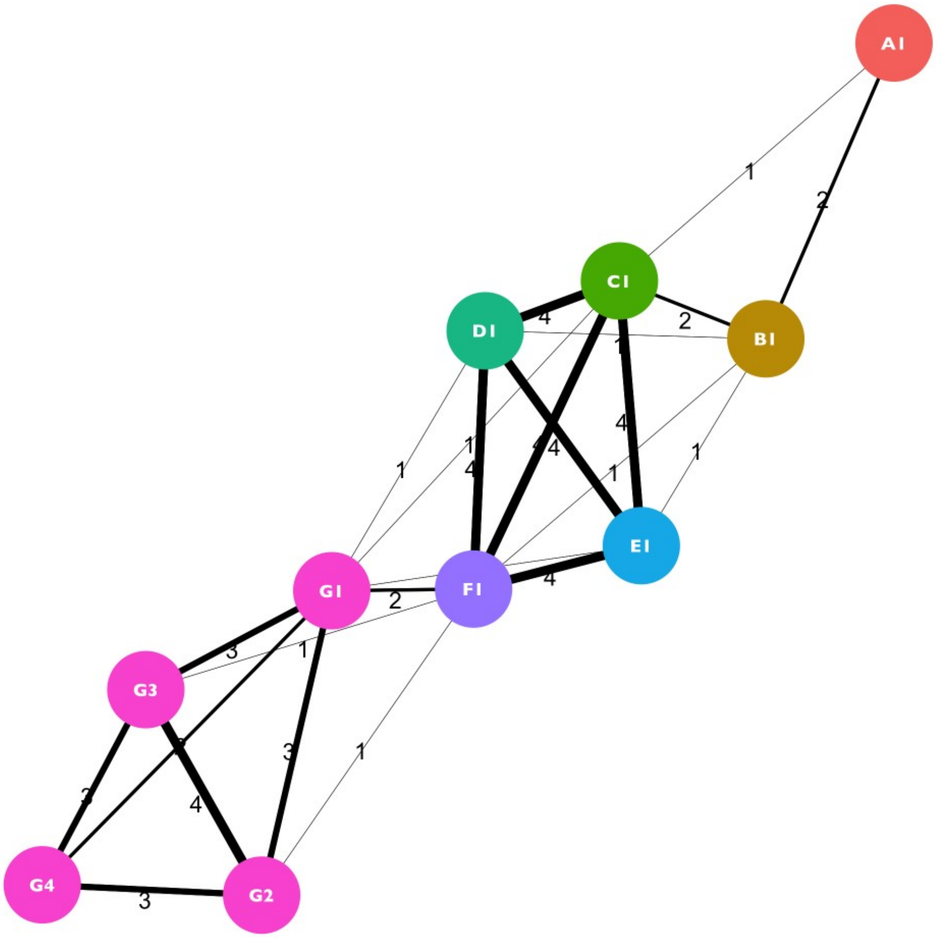

eExhibit 8: Unipartite Network for Example Network

Network Analytic Measures

The diagonal in the unipartite matrix depited in eExhibit 7 provides a count of the total number of in-network clinicians in each network. Alternatively, this value could be found by taking the column totals from the bipartite matrix depicted in eExhibit 6. The total number of in-network clinicians, divided by the total number of clinicians in the geographic market (i.e, the number of rows in the unipartite matrix in), is defined as the **breadth** of the network. That is, **network breadth** is the fraction of area physicians or hospitals that are in-network for a given plan network.

eExhibit 9 sizes the nodes by their breadth: larger nodes indicate broader provider networks. As seen here, **A1** is the most narrow network, since it has only 2 of the 10 providers in its network (breadth = 0.2 = 2/10).

© 2020 Graves JA et al. *JAMA Network Open.*

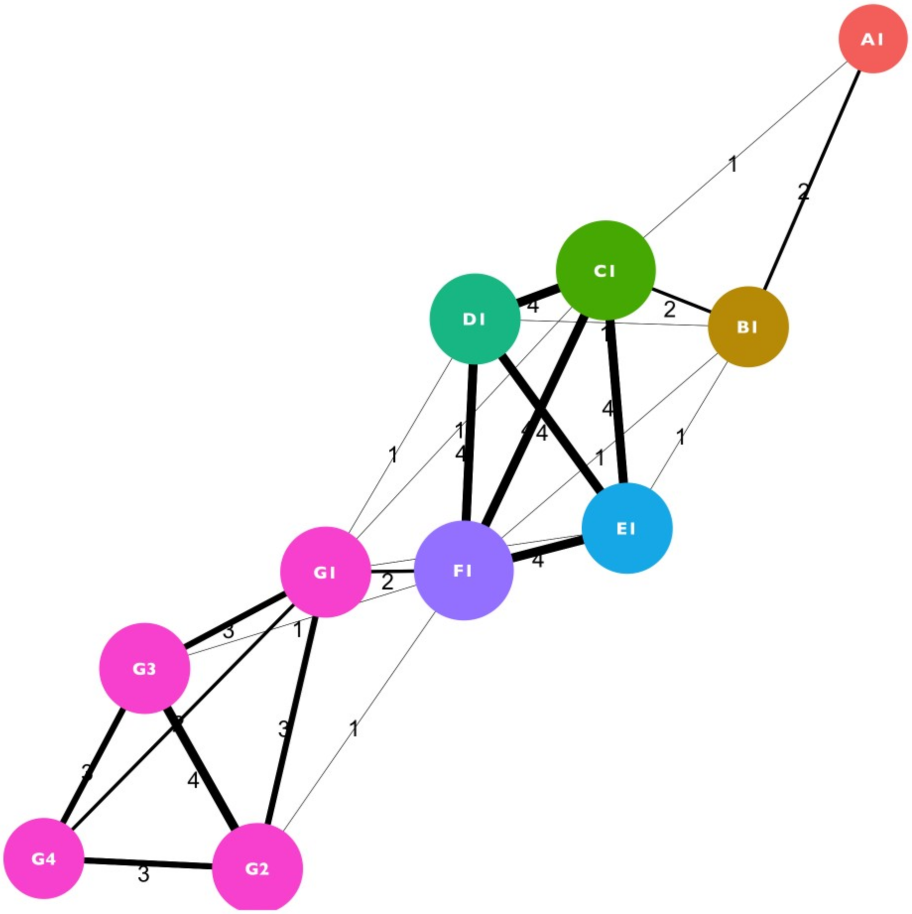

eExhibit 9: Unipartite Network for Example Network, With Node Size Scaled to Reflect Network Breadth

While the network breadth measure provides information on the overall size of a provider network, it does not capture information on how connected the provider network is to other networks in the area. That is, provider networks for two competing insurers could be relatively broad but each may have exclusive contracts with its in-network physicians and/or hospitals.

We thus construct a measure, the *normalized strength* of the network. This measure is defined as the total number of connections to other networks divided by the total possible of connections. Thus, a completely exclusive network (i.e., no connections with other insurers) will receive a value of 0, while a network with many connections will receive a value closer to 1.

It is easiest to build intuition for the normalized strength measure using the bipartite (i.e., provider-network) matrix. eExhibit 10 highlights how we measure strength for a single network (**G1**).

© 2020 Graves JA et al. *JAMA Network Open.*

Clinician ID	Network ID (Insurer-Network)									
	A1	B1	C1	D1	E1	F1	G1	G2	G3	G4
NPIA	1	1	0	0	0	0	0	0	0	0
NPIB	1	1	1	0	0	0	0	0	0	0
NPIC	0	1	1	1	1	1	0	0	0	0
NPID	0	0	1	1	1	1	0	0	0	0
NPIE	0	0	1	1	1	1	0	0	0	0
NPIF	0	0	1	1	1	1	1	0	0	0
NPIG	0	0	0	0	0	1	1	1	1	0
NPIH	0	0	0	0	0	0	1	1	1	1
NPII	0	0	0	0	0	0	1	1	1	1
NPIJ	0	0	0	0	0	0	0	1	1	1

There are 4 clinicians in network G1

There are 5 total connections between network G1 and other insurers' networks.

There are 24 opportunities for connections between network G1 and networks of other insurers.

Breadth: 100 * 4/10 = 40%

Exclusivity: 100 * 5 / 24 = 20.8%

eExhibit 10: Bipartite Matrix for Example

In this example we are interested in the degree to which a given insurer's network is connected to **other** insurers' networks. Thus, a count of the total number of shared connections with other insurers' networks (shown in red in eExhibit 10) is the numerator for measuring strength. In the case of network **G1** the total number of shared connections is 5.

The denominator for the normalized strength measure is a count of the total number of **possible** connections a given network could have with other networks. For the example network, there are 4 in-network clinicians and 6 networks offered by other insurers. Thus, if those 4 in-network clinicians for **G1** also are in-network for the other 6 networks, there are a total of 24 possible connections. Thus, the normalized strength value for network **G1** is 0.2083 (5/24).

Our primary results rely on strength measures as described above–that is, measures constructed by considering **only** connections with other insurers' networks. However, we also considered a total strength measure whereby we allowed for connections across all networks in the area.

eExhibit 11 below adds in our grouped strength ratio measure as hollow "rings" at each node. As can be seen in eExhibit 11, networks only loosely connected to other insurers' networks receive small normalized strength values.

© 2020 Graves JA et al. *JAMA Network Open.*

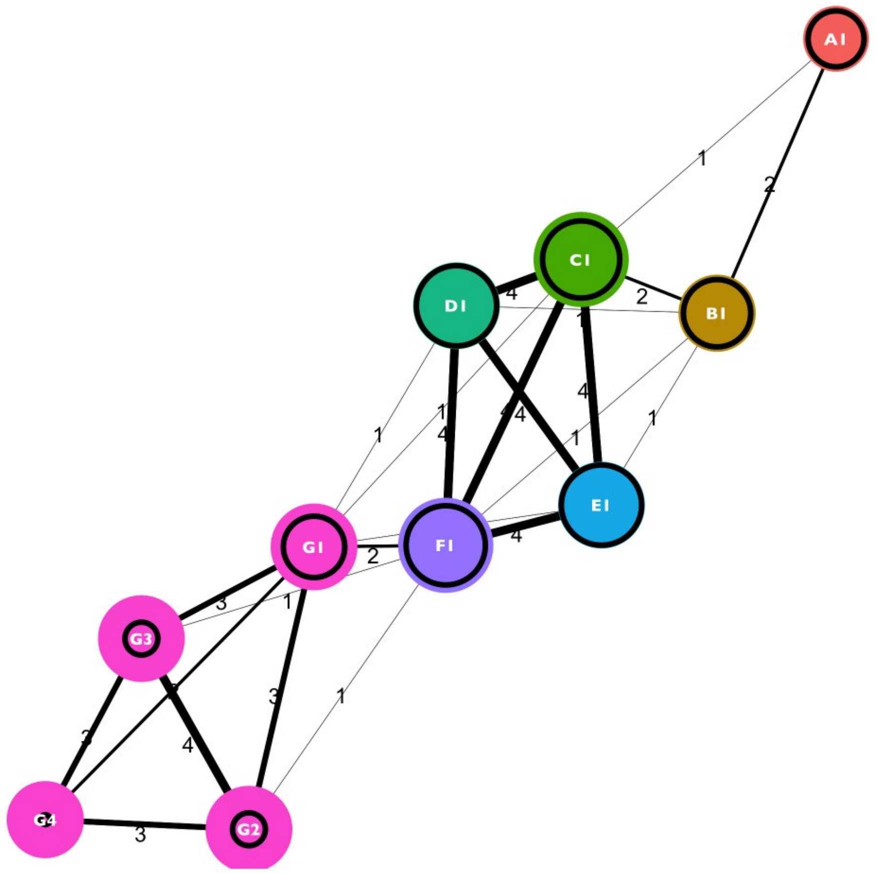

eExhibit 11: Unipartite Network for Example Network, With Node Size Scaled to Reflect Network Breadth and Grouped Strength Added

Network Analytic Measures for Example ZIP Network

We next show in eExhibit 12 a similar "network-of-networks" for the example ZIP code (53005). This network was constructed for cardiologists listed in large group insurance networks. We see, for example, that the United Navigate Plus network (pink node near center of figure) has a network breadth of 0.866, indicating that 86.6% of the 224 cardiologists within a 60-minute drive time of the ZIP centroid are in-network. By comparison, the Aetna Quality Point-of-Service (QPOS) plan (small red node at top of figure) is quite narrow, with only 6.7% of cardiologists in-network. Yet, despite being narrow the Aetna plan is quite well connected: it features 69.2% of all possible connections with other large-group insurers, while the much broader United Navigate Plus plan features 51.9% of all possible connections.

© 2020 Graves JA et al. *JAMA Network Open.*

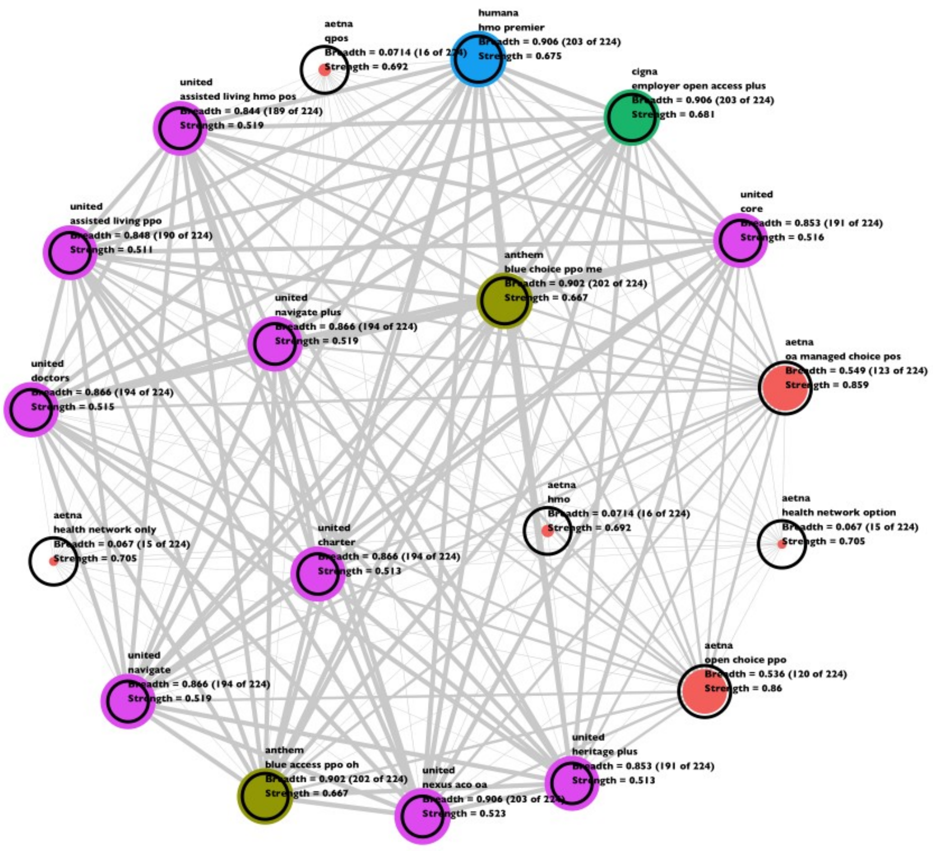

eExhibit 12: Primary Care Physician Network-of-Networks for Small Group Plans in Example ZIP Code

© 2020 Graves JA et al. *JAMA Network Open.*

Supplemental Results

			Percentile					
	No. Obs[1]	Mean (SD)	10th	25th	50th	75th	90th	Network Undefined[2]
All Connections								
Primary Care	1,248,138	60.9 (11.1)	49.7	55.3	60.6	67.7	74.0	1,515 [3.9]
Acute Care Hospital	1,446,413	63.5 (11.1)	51.5	57.2	63.4	69.6	76.9	454 [1.3]
Cardiology	1,173,486	65.7 (10.9)	52.7	58.5	65.8	73.0	79.2	4,189 [8.4]
Only Connections with Other Insurers								
Primary Care	1,248,138	56.5 (11.9)	43.1	49.7	56.6	64.0	71.0	1,515 [3.9]
Acute Care Hospital	1,446,413	59.6 (12.2)	45.7	52.4	59.7	66.6	74.2	454 [1.3]
Cardiology	1,173,486	62.2 (11.9)	48.0	54.5	61.9	70.4	77.3	4,189 [8.4]

[1] Unit of analysis is the ZIP-market-network-specialty.

[2] Number of ZIPs [population in millions] with no physician/hospital within a 60-minute travel time.

SD = Standard deviation

eExhibit 13: Sensitivity Analysis: Total Strength (allowing for all possible ponnections) vs. Grouped Strength (allowing only for connections with other insurers)

© 2020 Graves JA et al. *JAMA Network Open.*

	No. Obs[1]	Mean (SD)	Percentile					no_net[2]
			10th	25th	50th	75th	90th	
Network Breadth								
Primary Care	1,116,213	50.6 (21.9)	18.6	34.5	52.1	68.0	79.0	3,760 [25.2]
Acute Care Hospital	1,336,048	58.9 (27.2)	16.7	37.5	61.1	80.0	100.0	1,926 [21.9]
Cardiology	970,056	62.2 (25.0)	24.6	44.3	66.7	83.1	92.0	8,320 [36.6]
Network Exclusivity								
Primary Care	1,116,213	58.5 (12.2)	44.9	51.6	58.3	66.2	73.1	3,760 [25.2]
Acute Care Hospital	1,336,048	62.7 (14.4)	46.0	54.2	62.3	71.8	81.2	1,926 [21.9]
Cardiology	970,056	64.3 (12.3)	49.8	56.2	63.9	72.7	80.1	8,320 [36.6]

[1] Unit of analysis is the ZIP-market-network-specialty.

[2] Number of ZIPs [population in millions] with no physician/hospital within a 60-minute travel time.

SD = Standard deviation

Urban ZIP codes defined as those with population-weighted centroids within metropolitan core-based statistical areas (CBSAs). There are 19,702 such ZIPs in the U.S.

eExhibit 14: Sensitivity Analysis: 30-Minute Isochrone for Urban Areas

© 2020 Graves JA et al. *JAMA Network Open.*

			Percentile					
	No. Obs[1]	Mean (SD)	10th	25th	50th	75th	90th	Network Undefined[2]
**Network Breadth**								
Primary Care	1,248,138	48.3 (21.8)	16.4	31.5	49.5	65.6	77.0	1,515 [3.9]
Acute Care Hospital	1,446,413	55.4 (25.9)	14.3	36.4	58.6	75.9	87.1	454 [1.3]
Cardiology	1,173,486	59.5 (24.9)	22.9	40.0	63.3	80.6	89.6	4,189 [8.4]
**Network Exclusivity**								
Primary Care	1,248,138	56.5 (11.9)	43.1	49.7	56.6	64.0	71.0	1,515 [3.9]
Acute Care Hospital	1,446,413	59.6 (12.2)	45.7	52.4	59.7	66.6	74.2	454 [1.3]
Cardiology	1,173,486	62.2 (11.9)	48.0	54.5	61.9	70.4	77.3	4,189 [8.4]

[1] Unit of analysis is the ZIP-market-network-specialty.

[2] Number of ZIPs [population in millions] with no physician/hospital within a 60-minute travel time.

SD = Standard deviation

© 2020 Graves JA et al. *JAMA Network Open.*

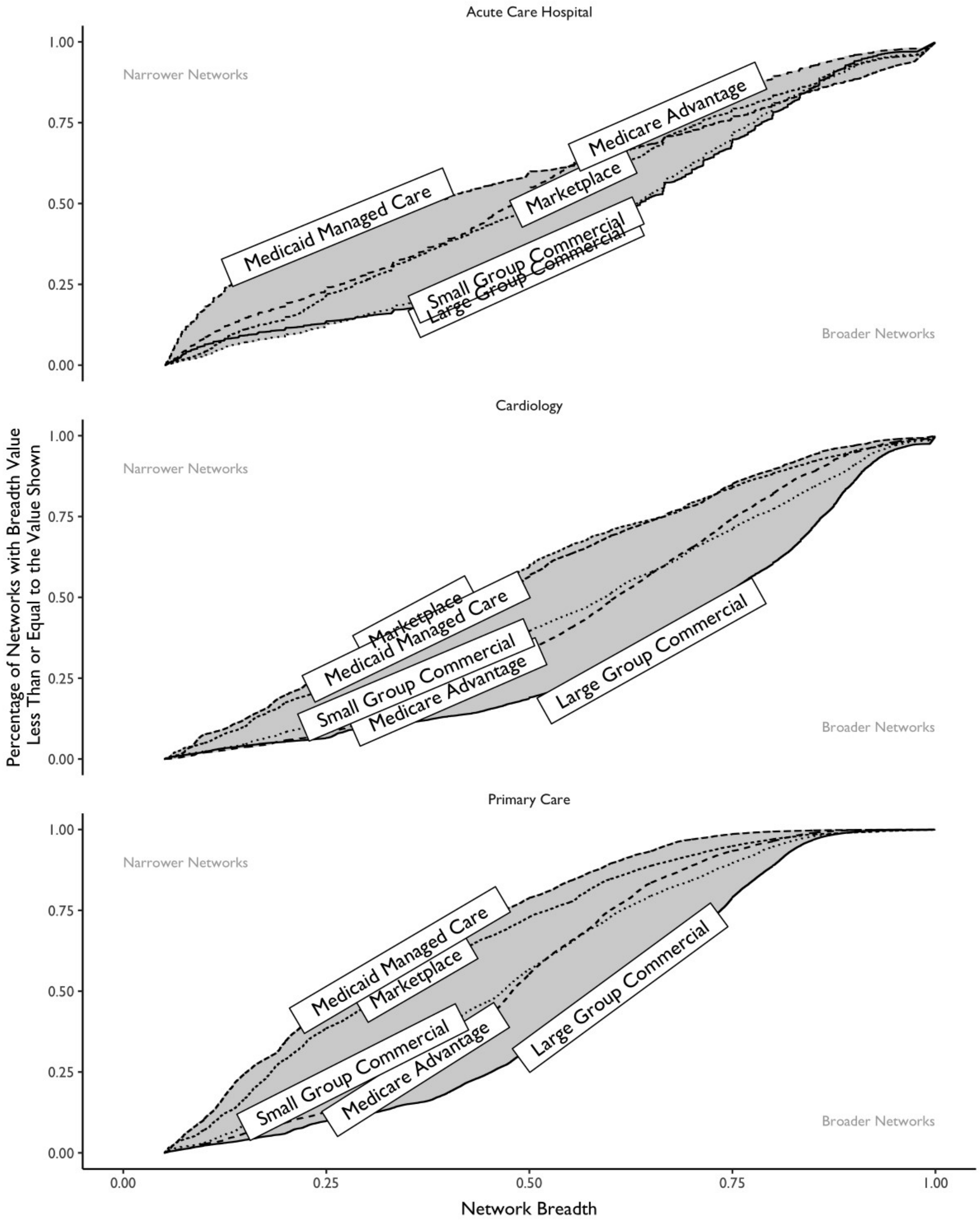

eExhibit 15: Distribution of Network Breadth by Market

© 2020 Graves JA et al. *JAMA Network Open.*

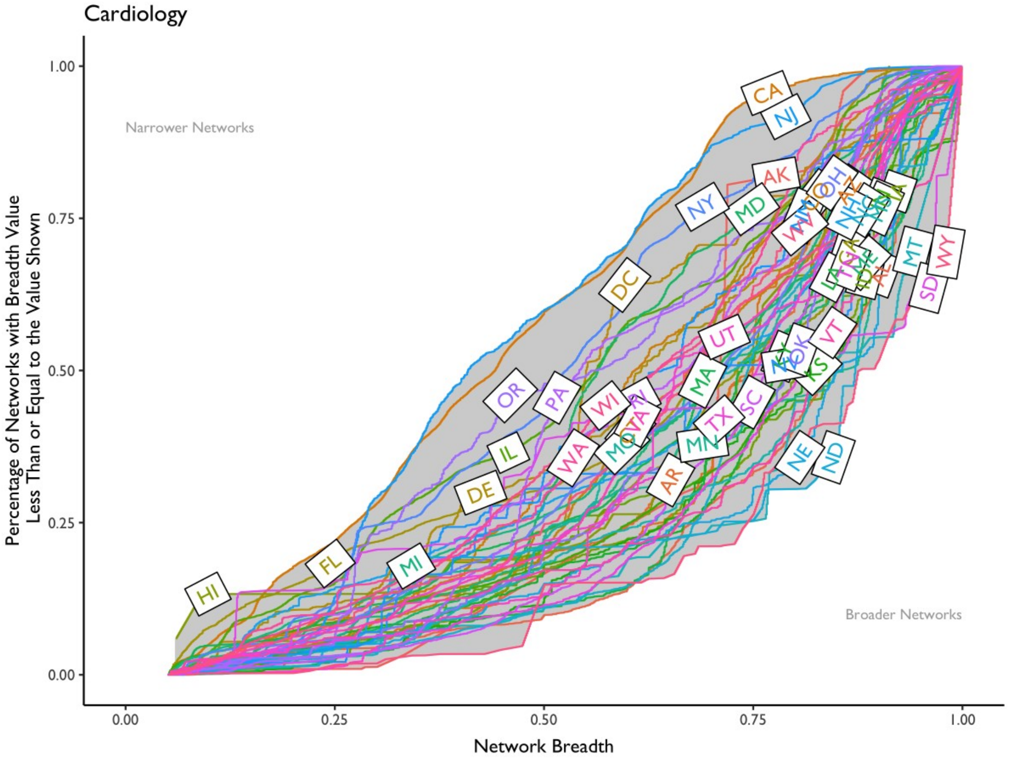

eExhibit 16: Empirical Cumulative Distribution of Network Breadth by State: Cardiology

© 2020 Graves JA et al. *JAMA Network Open.*

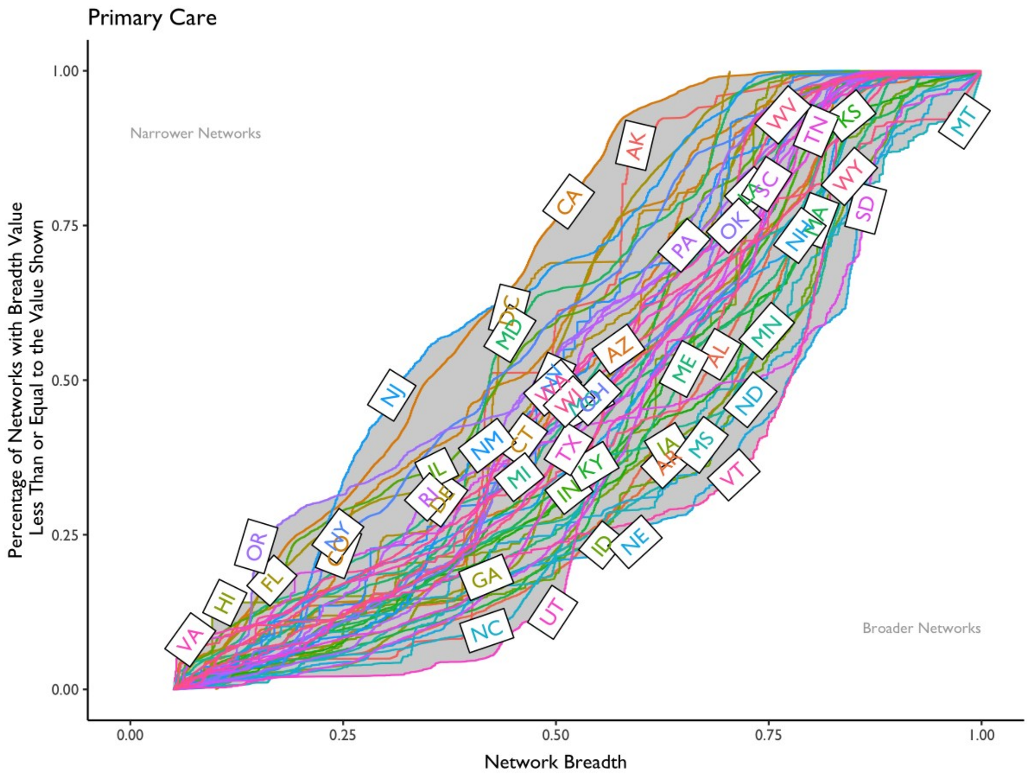

eExhibit 17: Empirical Cumulative Distribution of Network Breadth by State: Primary Care

© 2020 Graves JA et al. *JAMA Network Open.*

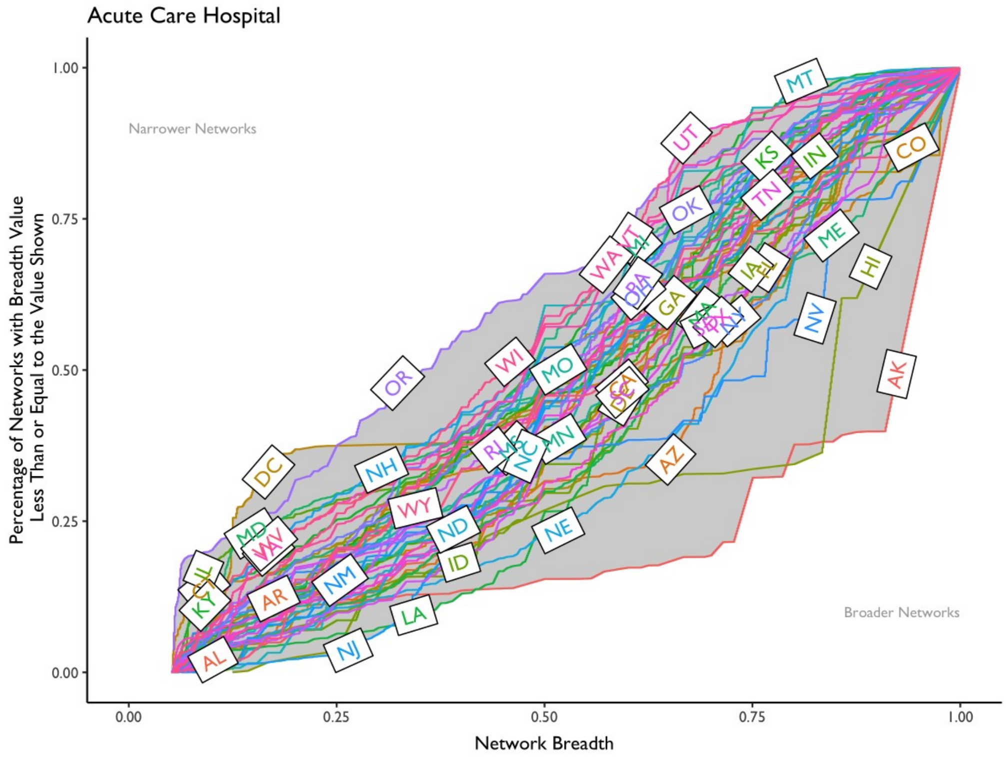

eExhibit 18: Empirical Cumulative Distribution of Network Breadth by State: Acute Care Hospital

#

© 2020 Graves JA et al. *JAMA Network Open.*

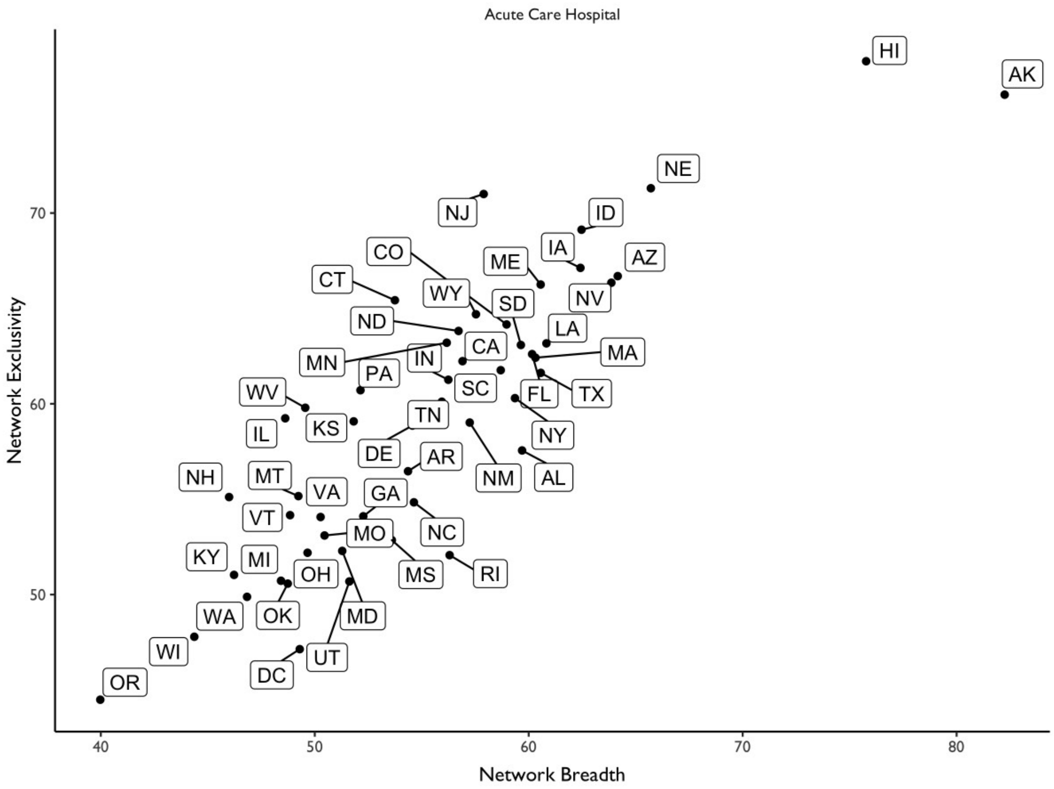

eExhibit 19: Average Network Breadth and Exclusivity by State: Acute Care Hospital

© 2020 Graves JA et al. *JAMA Network Open.*

			Mean Exclusivity by Network Breadth Size								Mean Exclusivity by Network Breadth Size				
State	Mean Breadth	Mean Exclusivity	Extra-Small	Small	Medium	Large	Extra-Large	State	Mean Breadth	Mean Exclusivity	Extra-Small	Small	Medium	Large	Extra-Large
AK	84.0	74.3	66.2	67.4	65.1	57.7	76.1	NC	60.4	53.3	41.7	48.5	56.7	56.0	52.3
AL	59.0	56.8	49.0	45.5	56.6	57.1	58.2	ND	55.2	64.0	53.8	56.6	50.6	67.8	67.2
AR	59.9	54.2	52.4	53.9	56.3	51.2	54.7	NE	71.8	71.2	62.9	76.1	70.6	74.8	70.6
AZ	65.4	64.9	61.0	60.1	59.2	61.5	67.0	NH	50.5	53.8	51.1	57.1	64.2	59.6	49.9
CA	57.6	63.2	66.6	64.9	63.6	63.8	62.1	NJ	70.9	64.7	88.1	81.1	74.3	66.7	62.6
CO	78.5	60.3	57.4	66.5	66.7	59.0	60.2	NM	51.9	61.5	58.4	62.4	62.8	62.2	59.6
CT	57.4	63.7	89.6	55.5	63.1	63.3	54.3	NV	67.5	59.9	54.6	55.6	55.7	47.5	62.6
DC	48.9	45.1	50.6	58.5		44.7	38.1	NY	73.8	56.7	62.4	63.4	52.8	60.1	56.5
DE	62.2	57.2	73.2	60.3	67.7	56.4	55.2	OH	52.4	51.1	54.0	55.4	50.7	52.0	49.4
FL	64.8	61.6	60.7	60.6	66.3	61.4	61.6	OK	55.5	48.5	25.8	53.3	54.6	52.0	48.2
GA	61.1	52.5	57.4	58.3	52.6	53.7	51.1	OR	59.8	49.5	18.0	55.6	59.6	50.8	49.5
HI	85.4	72.8		49.2	80.8	87.2	72.2	PA	53.8	60.6	77.0	74.3	67.1	59.2	54.7
IA	67.8	65.3	55.9	56.4	66.4	69.3	65.2	RI	60.7	48.9	59.5	58.7	55.3	57.1	40.5
ID	73.1	60.4	45.0	51.8	67.0	68.2	59.7	SC	64.7	59.5	21.6	38.6	63.0	62.9	61.1
IL	49.1	60.8	84.7	55.3	55.1	54.5	51.0	SD	62.9	62.4	69.5	64.4	62.4	61.3	61.8
IN	56.9	59.8	67.7	60.1	56.3	61.7	57.6	TN	54.2	58.2	53.8	60.1	62.9	61.1	55.2
KS	56.7	57.2	68.6	62.2	54.7	60.0	55.5	TX	64.3	61.8	59.0	63.5	72.3	64.9	60.0
KY	46.8	50.6	61.1	60.5	51.7	46.9	45.5	UT	54.4	50.7	46.3	50.2	46.0	49.2	59.9
LA	61.6	63.2	48.6	65.6	72.9	66.4	60.7	VA	57.7	51.9	44.7	61.0	57.5	53.5	49.7
MA	67.2	59.7	66.0	67.9	66.8	64.0	57.5	VT	51.8	55.0	68.7	47.7	59.6	55.7	54.9
MD	58.8	49.4	48.9	53.8	58.6	49.4	47.7	WA	53.1	51.8	45.9	58.4	52.5	50.0	52.6
ME	57.0	64.3	61.7	61.8	71.2	67.3	64.5	WI	45.5	47.3	53.0	49.4	49.5	47.0	44.3
MI	46.0	49.7	47.3	52.4	47.7	49.4	51.7	WV	50.2	59.6	56.4	68.7	67.1	58.0	56.6
MN	66.2	57.8	58.2	59.2	62.0	60.9	55.8	WY	57.8	65.1	60.6	84.3	68.5	66.6	54.9
MO	56.0	50.9	41.8	59.2	53.4	50.3	49.8								
MS	58.1	51.9	37.3	49.7	53.1	54.0	52.2								
MT	51.8	44.9	57.5	38.6	43.9	40.0	50.9								

eExhibit 20: Mean Breadth and Exclusivity of Large Group Hospital Networks

© 2020 Graves JA et al. *JAMA Network Open.*

State	Mean Breadth	Mean Exclusivity	Mean Exclusivity by Network Breadth Size					State	Mean Breadth	Mean Exclusivity	Mean Exclusivity by Network Breadth Size				
			Extra-Small	Small	Medium	Large	Extra-Large				Extra-Small	Small	Medium	Large	Extra-Large
AZ	51.4	67.8	69.8	66.7	68.6	71.5	67.5	NE	74.7	68.7		94.7	68.7	71.5	66.8
CA	26.2	60.5	58.2	60.3	62.6	61.8	64.8	NH	63.1	49.5			49.5	47.3	50.5
CO	62.3	61.8	53.2	61.9	58.3	66.1	64.4	NJ	37.5	78.0		76.4	80.3	75.3	71.9
DC	16.6	53.0	35.7	53.5				NM	89.9	59.0		75.8	67.2	64.6	58.4
DE	33.3	60.9	47.8	47.6	68.7	74.3	68.4	NY	24.5	65.2	59.7	69.3	76.1	61.7	68.0
FL	54.5	66.3	69.0	71.0	68.6	64.6	63.6	OH	37.8	56.4	57.8	58.8	58.3	51.2	52.3
GA	48.8	54.2	51.6	52.7	51.9	54.1	56.8	OR	24.6	50.2	48.0	50.3	42.7	69.6	77.2
HI	78.6	81.2		89.6	91.2	84.0	77.4	PA	58.3	55.1	63.6	58.6	56.5	55.0	53.7
IA	76.5	68.1			67.0	62.4	68.9	RI	36.5	59.5		59.8	59.0	59.4	62.3
IL	46.6	53.4	57.3	52.8	51.3	53.5	53.3	SC	42.9	62.3	70.5	60.5	63.5	60.9	61.5
IN	74.9	60.2	40.5	42.1	48.6	56.9	63.2	TN	79.7	60.6		65.7	66.3	64.8	59.8
KS	72.5	59.3	92.6	68.0	64.2	60.2	58.1	TX	34.5	65.9	74.9	59.7	60.1	61.7	63.9
KY	17.4	60.5	62.8	63.0	47.4	44.8	42.5	UT	11.6	57.5	53.2	59.2	79.3	99.5	64.0
LA	77.9	62.8		25.1	66.4	62.9	62.7	VA	64.2	57.1	55.5	60.8	59.1	57.2	55.9
MA	69.6	60.6		76.5	62.9	60.7	60.3	WA	61.5	49.3	38.8	40.6	62.8	66.5	50.0
MD	34.3	53.6	68.8	52.7	54.2	53.2	52.8	WI	39.5	48.0	47.0	50.9	45.7	47.2	47.3
MI	34.5	51.0	54.9	45.8	51.0	50.1	53.3	WV	30.1	53.6	55.0	63.2	48.0	49.8	51.4
MN	10.2	68.3	68.4	67.5	75.3	74.3									
MO	62.0	56.6	88.5	66.0	60.5	54.6	55.2								
MS	52.2	52.7	65.4	50.1	48.9	51.2	56.9								

eExhibit 23: Mean Breadth and Exclusivity of Medicaid Managed Care Hospital Networks

© 2020 Graves JA et al. *JAMA Network Open.*

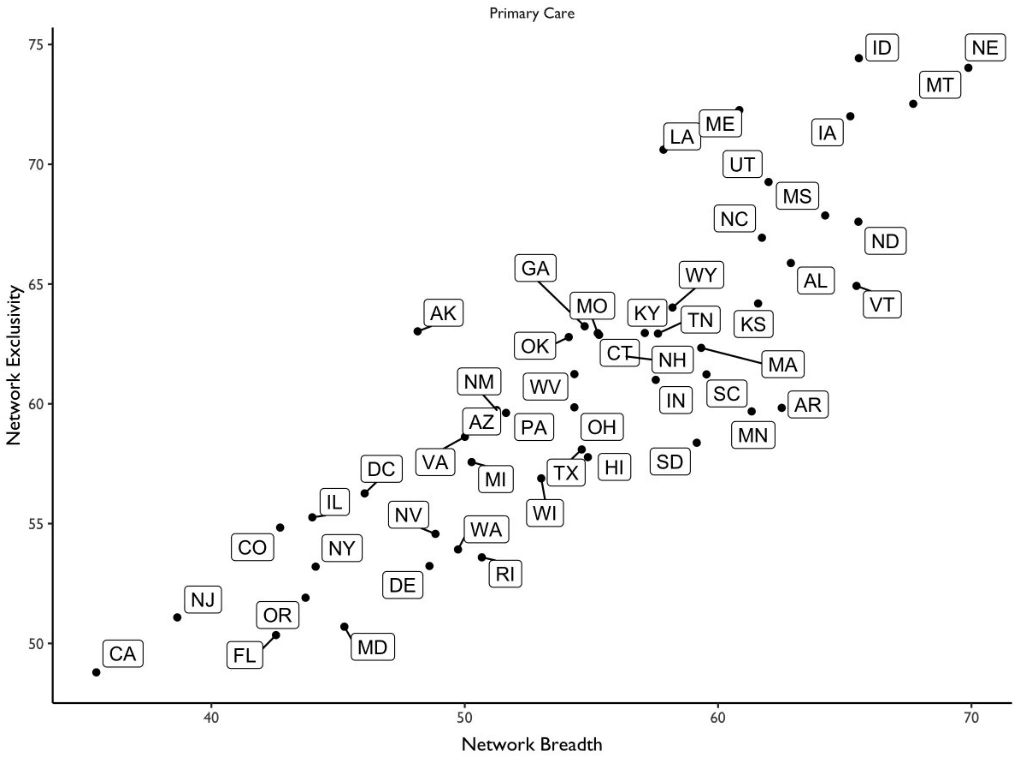

eExhibit 25: Average Network Breadth and Exclusivity by State: Primary Care Networks

© 2020 Graves JA et al. *JAMA Network Open.*

			Mean Exclusivity by Network Breadth Size								Mean Exclusivity by Network Breadth Size				
State	Mean Breadth	Mean Exclusivity	Extra-Small	Small	Medium	Large	Extra-Large	State	Mean Breadth	Mean Exclusivity	Extra-Small	Small	Medium	Large	Extra-Large
AK	47.1	61.0	50.0	47.5	67.1	64.6	30.8	NC	48.2	74.8	77.3	77.4	68.1	75.0	71.0
AL	67.8	66.8	67.3	59.2	67.1	69.2	66.2	ND	46.5	69.2	61.9	78.1	56.7	76.2	67.4
AR	71.6	58.4		63.2	65.6	68.1	56.6	NE	63.8	77.6	82.6	81.1	80.3	77.8	76.9
AZ	54.1	61.5	68.1	56.1	78.4	59.3	65.8	NH	50.7	69.5		70.6	70.8	72.8	66.7
CA	34.8	49.9	38.2	48.6	52.2	50.0	47.8	NJ	38.2	54.4		55.9	55.6	52.7	51.9
CO	41.2	53.3	50.5	46.2	56.3	57.5	54.7	NM	49.7	64.3	60.2	67.5	63.4	63.4	65.0
CT	46.1	71.3	73.2	72.2	71.5	72.3	68.3	NV	50.4	57.1	72.4	63.2	58.5	58.7	49.7
DC	41.3	59.3			59.9	59.0		NY	40.6	55.9	59.9	55.7	58.7	55.8	54.0
DE	36.8	57.5		54.2	55.1	62.3	60.6	OH	59.5	58.0	66.3	63.9	64.2	62.9	54.4
FL	46.5	50.2	49.5	57.9	57.6	48.9	46.2	OK	52.5	64.8	64.3	70.9	66.8	67.6	60.7
GA	54.7	64.2	65.2	64.8	62.0	65.7	64.0	OR	45.2	51.8	57.7	30.1	60.0	59.0	62.3
HI	50.8	53.1	17.7	4.9	54.7	54.9	67.3	PA	48.5	62.0	60.1	65.3	61.5	59.0	68.4
IA	54.0	76.3	67.4	72.0	69.5	78.6	78.8	RI	44.2	59.0	57.5	63.8	61.5	56.3	55.6
ID	65.0	77.0	74.3	75.7	76.1	76.5	77.4	SC	65.3	59.9	61.0	63.8	59.5	62.8	58.6
IL	35.9	60.5	72.2	63.2	58.7	55.9	56.6	SD	60.6	58.4	77.0	66.5	62.1	65.1	52.8
IN	56.4	61.9	62.1	67.0	67.6	62.5	59.1	TN	65.7	63.8		74.1	62.7	64.0	63.7
KS	63.6	66.8	67.7	73.4	66.2	66.9	66.8	TX	61.2	57.0	60.5	51.0	57.1	61.1	55.9
KY	62.2	62.4	64.1	66.9	69.2	68.8	59.2	UT	61.5	71.5	79.9	90.3	75.5	72.1	70.7
LA	57.9	72.5	74.4	74.8	73.5	72.7	72.0	VA	45.6	57.7	7.6	65.8	58.3	62.7	68.5
MA	72.4	61.0	63.7	62.6	61.8	58.2	61.1	WA	48.2	55.1	67.2	47.5	64.0	56.4	52.2
MD	38.5	55.0	19.9	60.7	56.3	55.1	69.0	WI	52.5	57.8	64.7	57.4	59.3	59.9	55.4
ME	62.4	76.2	31.0	73.6	74.2	77.0	76.7	WV	51.4	66.4	65.3	63.6	58.5	68.0	66.3
MI	51.4	58.8	57.5	62.2	63.4	58.7	56.8	WY	70.1	59.8		55.6	46.1	72.6	59.8
MN	55.6	62.2	67.1	62.9	65.7	68.8	60.6								
MO	45.9	68.9	72.7	72.2	71.8	67.9	66.1								
MS	75.1	71.1				65.2	71.2								
MT	68.5	73.7	80.6	65.2	63.9	76.3	75.6								

eExhibit 27: Mean Breadth and Exclusivity of Small Group Primary Care Networks

© 2020 Graves JA et al. *JAMA Network Open.*

			Mean Exclusivity by Network Breadth Size								Mean Exclusivity by Network Breadth Size				
State	Mean Breadth	Mean Exclusivity	Extra-Small	Small	Medium	Large	Extra-Large	State	Mean Breadth	Mean Exclusivity	Extra-Small	Small	Medium	Large	Extra-Large
AK	72.2	73.2					73.2	NC	65.2	79.5	70.4	77.2	84.1	75.4	79.6
AL	83.5	75.2	68.7	71.0	72.5	74.3	75.4	ND	48.5	59.7		40.9	68.0		56.6
AR	75.4	63.5	62.2	66.5	66.8	69.8	62.4	NE	76.0	77.0		91.7	85.3	78.0	76.5
AZ	71.7	73.3	81.7	79.3	80.5	76.8	72.4	NH	58.6	76.8		80.5	81.0	79.3	73.7
CA	43.4	52.6	37.4	54.5	54.9	53.0	51.1	NJ	45.0	58.7		59.9	59.2	58.6	57.7
CO	59.3	67.5	47.1	63.2	68.2	68.6	67.6	NM	56.7	72.2	75.0	74.8	72.1	69.0	73.3
CT	56.8	74.4	82.6	75.7	72.2	76.8	73.2	NV	72.0	64.5	86.7	71.6	75.1	67.5	62.7
DC	55.3	63.3				63.3		NY	49.8	57.3	60.0	58.2	55.5	58.5	57.1
DE	43.7	66.5		64.1	59.1	69.0	73.6	OH	69.5	63.3	72.7	70.1	70.1	66.3	61.0
FL	67.5	56.7	62.5	66.9	66.1	58.6	55.3	OK	66.9	70.4	54.8	79.0	67.0	70.3	69.7
GA	71.2	70.3	58.2	69.4	69.3	72.9	70.2	OR	56.9	59.6	75.6	59.4	52.4	62.5	62.6
HI	59.8	51.5	17.3			55.5	62.8	PA	56.6	64.8	36.3	69.2	64.6	60.2	71.7
IA	65.3	78.4	80.5	69.4	74.6	73.9	81.2	RI	49.8	68.7		72.9	71.6	68.5	64.8
ID	70.7	72.9	74.2	83.7	68.3	72.0	73.9	SC	77.8	71.1	92.6	76.7	68.3	72.9	70.7
IL	48.4	66.2	69.7	72.8	70.8	62.9	60.8	SD	68.4	58.7	70.0	77.6	58.6	63.4	54.0
IN	70.6	70.1	72.1	70.2	74.1	74.5	68.7	TN	81.6	74.8		74.7	77.3	76.6	74.6
KS	78.2	77.5	77.6	88.4	75.8	76.9	77.6	TX	74.6	63.7	71.6	61.6	61.3	66.7	63.5
KY	79.0	68.6	82.5	65.2	68.4	66.9	68.7	UT	66.4	72.3		73.8	71.4	70.7	72.9
LA	70.6	80.8		81.0	85.2	85.0	80.4	VA	66.8	71.8	79.1	78.3	77.0	68.1	72.4
MA	72.3	68.1	70.1	71.4	69.9	68.0	67.7	WA	56.5	63.0	68.7	68.4	48.2	62.3	63.6
MD	50.9	63.1	44.5	64.1	63.4	63.1	67.0	WI	63.6	63.3	66.2	68.0	66.4	66.4	60.7
ME	71.9	78.1	60.0	72.5	78.4	80.7	77.2	WV	63.7	66.4		73.4	67.2	62.1	67.7
MI	66.1	67.4	65.9	67.7	69.4	68.7	67.0	WY	87.6	74.9				33.3	83.0
MN	63.1	68.0	81.4	68.7	71.4	70.3	64.5								
MO	62.0	76.7	92.6	77.9	81.7	76.8	75.0								
MS	90.4	72.1					72.1								
MT	79.6	68.7			70.1	49.2	71.5								

eExhibit 33: Mean Breadth and Exclusivity of Small Group Cardiology Networks

© 2020 Graves JA et al. *JAMA Network Open.*

State	Mean Breadth	Mean Exclusivity	Mean Exclusivity by Network Breadth Size					State	Mean Breadth	Mean Exclusivity	Mean Exclusivity by Network Breadth Size				
			Extra-Small	Small	Medium	Large	Extra-Large				Extra-Small	Small	Medium	Large	Extra-Large
AK	80.3	73.1					73.1	NC	61.0	77.2	59.3	74.0	80.8	78.6	75.5
AL	81.1	75.9	67.8	80.9	84.9	71.0	74.9	ND	73.7	57.8	35.8	40.3	74.2	55.1	56.8
AR	58.8	69.0	54.4	57.5	65.0	69.5	69.7	NE	75.1	74.6	19.1	56.9	73.4	76.4	74.2
AZ	44.1	76.4	56.6	78.8	77.2	75.7	75.6	NH	37.3	77.5		74.2	77.4	79.9	82.0
CA	36.1	50.2	13.4	51.5	55.4	56.2	51.3	NJ	40.8	59.2		58.5	58.3	59.1	62.6
CO	41.0	69.1	47.4	64.5	71.7	69.5	76.2	NM	57.2	72.1	51.5	76.6	72.0	71.6	73.1
CT	50.3	75.3	82.4	75.3	74.5	75.5	75.1	NV	58.0	65.6	66.2		65.6	65.6	65.6
DC	55.9	62.1				62.1		NY	42.7	57.5	59.3	58.1	55.0	55.9	62.3
DE	42.8	68.1		64.1	64.5	64.3	73.7	OH	38.0	68.9	66.9	70.2	69.7	67.0	69.0
FL	21.2	64.3	62.0	66.1	66.4	60.2	75.5	OK	66.8	69.7	43.1	66.2	66.1	70.1	70.9
GA	43.7	71.3	71.7	70.3	70.1	71.5	75.9	OR	44.5	57.2		59.1	52.2	62.5	62.4
HI	56.1	49.8	17.3			55.5	63.2	PA	51.5	64.6	46.6	62.8	70.3	60.0	74.1
IA	65.7	77.3	77.6	68.8	78.5	73.8	78.9	RI	37.2	71.6		73.5	72.6	69.8	72.7
ID	63.2	73.3	95.2	85.4	68.9	72.2	74.5	SC	80.8	72.9		78.3	70.7	70.6	73.0
IL	30.3	70.1	64.8	71.7	71.1	64.9	67.5	SD	40.6	58.8	70.0	73.3	58.6	58.4	63.1
IN	53.5	74.4	68.4	72.6	72.8	74.1	75.4	TN	60.2	77.3	86.5	80.4	80.4	76.9	75.7
KS	71.0	78.6	77.6	80.7	74.2	74.0	80.8	TX	46.5	68.9	72.2	70.9	67.5	67.8	70.6
KY	65.1	72.2	83.4	65.1	68.2	66.5	74.1	UT	57.0	72.7	80.0	70.8	70.8	72.1	73.8
LA	74.8	80.3		79.2	80.3	81.9	80.3	VA	64.6	70.8	77.9	81.7	79.4	65.0	71.5
MA	53.3	72.2	76.6	73.6	74.0	74.2	70.3	WA	43.8	62.2	62.4	55.3	56.1	65.9	70.8
MD	49.6	62.2	44.5	64.1	64.4	62.2	64.6	WI	45.3	64.6	61.7	63.3	57.3	66.3	68.4
ME	83.2	75.8		76.7	77.6	76.6	75.8	WV	48.2	70.0	77.1	54.9	69.4	69.9	74.8
MI	56.6	67.4	68.4	70.9	69.0	66.5	66.3	WY	77.4	72.9				18.7	84.4
MN	63.2	68.0	71.7	74.1	71.6	67.1	65.6								
MO	40.6	74.7	81.9	79.3	74.0	74.4	76.6								
MS	48.8	73.5	71.1	76.5	70.1	74.9	74.4								
MT	78.7	68.7			70.1	53.1	72.0								

eExhibit 34: Mean Breadth and Exclusivity of Marketplace Cardiology Networks

© 2020 Graves JA et al. *JAMA Network Open.*

			Mean Exclusivity by Network Breadth Size								Mean Exclusivity by Network Breadth Size				
State	Mean Breadth	Mean Exclusivity	Extra-Small	Small	Medium	Large	Extra-Large	State	Mean Breadth	Mean Exclusivity	Extra-Small	Small	Medium	Large	Extra-Large
AK	10.0	15.4	15.4					NC	72.7	73.9	84.3	81.1	76.6	74.8	73.0
AL	74.0	76.3	71.6	78.3	85.0	79.1	75.2	ND	86.3	56.1		57.0		63.1	56.1
AR	61.4	63.9	68.3	73.3	66.6	69.0	61.4	NE	52.0	75.7	81.6	79.3	76.5	80.3	70.1
AZ	66.8	73.3		82.1	70.7	77.3	71.7	NH	51.3	69.1	79.6	79.1	74.3	76.3	60.6
CA	42.2	54.7	60.3	57.6	55.3	53.6	53.7	NJ	40.7	58.7	70.1	60.4	58.7	59.8	53.5
CO	63.5	68.9	60.9	71.3	72.1	66.8	70.6	NM	51.9	69.7	78.9	69.6	70.1	69.2	69.4
CT	64.6	71.8	75.2	75.6	66.1	68.0	71.9	NV	32.5	62.3		70.6	61.8	63.3	74.6
DC	57.3	58.6				66.5	50.7	NY	53.6	55.5	59.9	59.8	55.6	55.4	54.2
DE	52.8	62.8	64.6	46.9	59.0	65.9	65.0	OH	64.2	66.2	69.0	67.7	67.6	67.9	65.5
FL	51.2	57.1	64.8	62.1	62.9	55.3	54.9	OK	66.0	70.1	79.6	66.2	69.4	70.5	70.2
GA	62.2	69.8	71.5	72.9	74.3	66.4	71.3	OR	51.9	58.0		43.8	54.7	64.9	59.4
HI	55.8	60.5			60.7	63.0	58.9	PA	54.7	61.2	64.2	54.0	60.2	60.1	64.0
IA	72.5	78.4	70.2	75.9	77.2	80.7	78.1	RI	68.8	56.5		75.0	73.5	70.2	53.2
ID	74.6	67.6		61.8	69.6	71.5	66.7	SC	66.8	72.8	78.0	77.4	71.5	74.0	72.4
IL	58.4	61.0	69.9	63.0	67.3	63.2	57.0	SD	66.6	61.7	75.9	69.4	41.0	69.5	61.4
IN	73.4	69.8	75.8	73.6	71.7	71.9	69.2	TN	75.1	75.4	68.5	74.1	79.0	72.1	75.5
KS	73.4	75.0	73.7	75.6	77.3	76.4	74.7	TX	67.7	63.9	65.8	66.7	71.5	67.4	62.1
KY	74.2	68.9	73.5	65.5	69.4	68.1	69.0	UT	61.6	70.3		69.5	73.1	71.4	68.2
LA	66.9	79.9	79.5	76.9	74.1	77.7	81.2	VA	55.2	72.0	79.8	71.3	77.0	69.7	72.5
MA	49.3	66.3	74.3	77.7	71.5	68.5	58.7	VT	74.1	67.7	57.6	55.7	58.5	67.1	69.5
MD	61.2	55.7	5.4	65.2	60.4	57.8	54.8	WA	58.9	61.2	66.0	61.2	50.6	66.0	60.7
ME	68.0	75.2		52.2	70.1	78.7	75.7	WI	64.7	61.0	64.3	61.5	62.0	64.8	58.8
MI	60.3	66.2	59.1	64.0	65.3	69.5	65.7	WV	61.7	65.2	67.7	61.7	62.4	64.2	66.4
MN	81.6	65.0		76.8	71.6	67.3	64.2	WY	65.3	86.4		84.2	86.1	87.3	86.4
MO	72.0	70.5	76.2	65.1	67.0	74.7	69.8								
MS	56.9	70.2	81.9	77.3	70.7	68.7	70.0								
MT	71.4	65.3	56.0	62.2	56.6	61.1	67.5								

eExhibit 36: Mean Breadth and Exclusivity of Medicare Advantage Cardiology Networks

© 2020 Graves JA et al. *JAMA Network Open.*
